# Novel Strategy for Optimized Nanocatalytic Tumor Therapy: From an Updated View

**DOI:** 10.1002/smsc.202200024

**Published:** 2022-06-03

**Authors:** Zhen-Li Li, Han Wu, Jia-Qi Zhu, Li-Yang Sun, Xiang-Min Tong, Dong-Sheng Huang, Tian Yang

**Affiliations:** ^1^ Department of General Surgery, Cancer Center, Division of Hepatobiliary and Pancreatic Surgery Zhejiang Provincial People's Hospital (People's Hospital of Hangzhou Medical College) Hangzhou Zhejiang 310014 China; ^2^ School of Public Health Hangzhou Medical College Hangzhou Zhejiang 310014 China; ^3^ Department of Hepatobiliary Surgery Eastern Hepatobiliary Surgery Hospital Second Military Medical University (Naval Medical University) Shanghai 200438 China; ^4^ Eastern Hepatobiliary Clinical Research Institute Third Affiliated Hospital of Naval Medical University Shanghai 200438 China; ^5^ College of Biotechnology and Bioengineering Zhejiang University of Technology Hangzhou Zhejiang 310014 China

**Keywords:** cancer therapy, nanocatalytic therapy, nanozymes

## Abstract

Nanozyme has been experiencing rapid development in biomedical applications involving biosensors, immunoassays, and antitumor agents in recent years due to its tunable catalytic performance and desirable biocompatibility. Since the first exploration of nanozyme‐based Fenton reaction for nanocatalytic therapy (NCT) against tumor, a variety of Fenton (and Fenton‐like) nanozymes, such as Fe_3_O_4_, transition metal ions (Co^2+^, Cu^2+^, and Mn^2+^), and metal–organic frameworks (MOFs), have been proved as desirable candidates for tumor therapy, and the modulation of the tumor microenvironment (TME) is determined to be a feasible approach to improve the catalytic efficiency for in situ tumor suppression. At present, increasing studies have focused on improving the therapeutic efficiency of NCT by formulating multifunctional nanozyme‐based systems to satisfy the demand for versatile and optimized applications. Herein, updated insights into the novel strategies of 1) achieving highly effective nanocatalytic reactions, including the modification of nanocatalysts and TME‐modulating approaches, are provided and 2) the design and formulation of multifunctional nanozyme‐based systems which achieve targeted, synergistic therapy, and theranostic applications are analyzed and concluded. Concise and concentrated comments and outlooks are illuminated at the end to outline the perspectives and the remaining challenges for the next‐step explorations on further biomedical translation of NCT.

## Graphical abstract

With the rapid development of nanocatalytic tumor therapy, the review provides updated insights into the novel strategies of optimized nanocatalytic therapy in the following aspects: 1) achieving highly effective nanocatalytic reactions, including the modification of nanocatalysts and tumor microenvironment‐modulating approaches and 2) the design and formulation of multifunctional nanozyme‐based systems which achieve targeted, synergistic therapy, and theranostic applications.

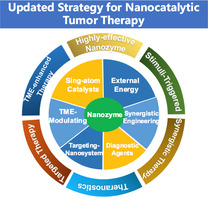

## 1 Introduction

Cancer management has been going through an era of coexisting advances and challenges. On the one hand, various targeted, molecular, and immune drugs with remarkable antitumor capability have been under research and applied to clinical practice, which bring about tremendous improvement on the prognosis of cancer therapy.^[^ ^1^ ^]^ On the other hand, the conventional strategies of medication with intrinsic toxicity and off‐target effects are still considered as insurmountable barriers.^[^ ^2^ ^]^ In this context, novel TME‐specific therapies with reduced side effects and improved antitumor performance have inspired great research interest in the scientific community.^[^ ^3^ ^]^ TME is characterized by mild acidosis and elevated H_2_O_2_ level as the result of the accelerated metabolism of cancer tissues and inadequate blood supply, reducing condition since the intratumor glutathione (GSH) level is about fourfold that in normal tissues.^[^ ^4^ ^]^ Such unique TME properties enable specific chemical/medical antitumor reactions in situ, avoiding off‐target effects of traditional medicine, which provide a novel concept on cancer management.^[^ ^5^ ^]^

**Scheme 1:**
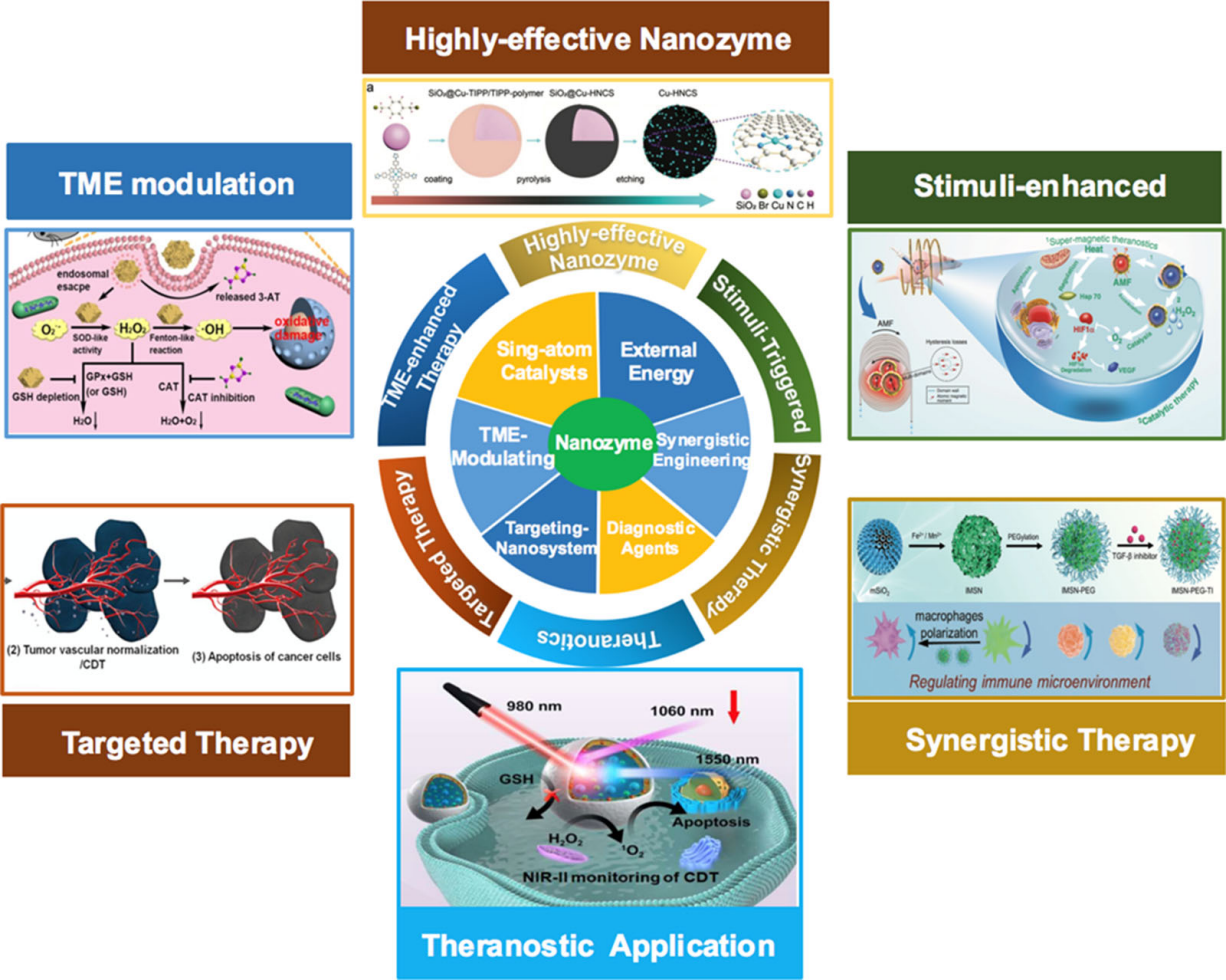
Schematic illustration of the various strategies for the enhanced NCT against cancer. Highly‐effective Nanoenzyme: Reproduced with permission.^[^ ^29^ ^]^ Copyright 2020, Wiley‐VCH; TME Modulation: Reproduced with permission.^[^ ^36^ ^]^ Copyright 2020, American Chemical Society; Targeted Therapy: Reproduced with permission.^[^ ^43^ ^]^ Copyright 2020, the American Association for the Advancement of Science; Stimuli‐enhanced: Reproduced under the terms of the CC‐BY 4.0 license.^[^ ^48^ ^]^ Copyright 2020, The Authors, published by Springer Nature. Synergistic Therapy: Reproduced with permission. Copyright 2020, Wiley‐VCH.^[^ ^53^ ^]^ Theranostic Application: Reproduced with permission.^[^ ^59^ ^]^ Copyright 2021, Wiley‐VCH.

The representative strategy taking advantages of TME features is the nanozyme‐mediated catalytic therapy on the basis of functional nanomaterials.^[^ ^6^ ^]^ Since the first demonstration of peroxidase‐like (POD) activity of Fe_3_O_4_ nanoparticles (NPs) in 2007,^[^ ^7^ ^]^ numerous NPs (nanozymes) have been proved to own intrinsic enzyme‐like abilities.^[6c,e,f]^ Among them, a variety of Fenton (and Fenton‐like) nanozymes have been utilized for chemodynamic therapy (CDT) or NCT.^[^ ^8^ ^]^ These nanozymes intrinsically possess excellent catalytic performance, small sizes, and large surface areas, facilitating the catalytic chemical reactions with the exposure of active sites.^[^ ^9^ ^]^ Owing to the specific TME with overproduced H^+^ and H_2_O_2_, the nanozyme would trigger reactive oxygen species (ROS) in situ, inducing necrosis and apoptosis of tumors.^[^ ^10^ ^]^ Since the initial application of Fe_3_O_4_ NPs for NCT in 2016, the investigation and progress of NCT could be summarized in following steps in recent years: 1) exploration of a variety of biocompatible high‐performance nanocatalysts for NCT, such as transition metal ions (Co^2+^, Cu^2+^, and Mn^2+^)^[^ ^11^ ^]^ and metal–organic frameworks (MOFs)^[^ ^12^ ^]^ which share parallel catalytic properties of Fe_3_O_4_ and 2) modulating TME conditions to maximize the catalytic reaction and generate elevated ROS for the improvement of NCT effects.^[^ ^13^ ^]^ 3) At current stage, besides the investigations on the above two aspects, increasing studies have focused on improving the therapeutic efficiency of NCT by formulating multifunctional nanozyme‐based systems to satisfy the demand for versatile applications, including targeted delivery of catalytic nanosystems^[^ ^14^ ^]^ and synergistic therapy combining latest immune,^[^ ^15^ ^]^ gene editing,^[^ ^16^ ^]^ and molecular medicine.^[^ ^17^ ^]^ Moreover, accurate theranostic nanoplatforms were successfully constructed to achieve real‐time detection of the antitumor effect of NCT^[^ ^18^ ^]^ **(** **Scheme** **1** **).**

There are several high‐quality reviews summarizing the characteristics and progress of NCT on biomedical applications.[^8^, ^19^] Yang et al^[8a]^ first proposed the concept of “nanocatalytic medicine” defined as “catalytic reaction‐based diagnosis and therapy using biocompatible nanomaterials”. Following reviews by Tang et al.^[19e]^ and Zhang et al.^[19b]^ concluded the various types of nanocatalysts for tumor therapy, and the TME‐modulating approaches were also listed and analyzed. Though comprehensive summary and progress of feasible nanocatalysts utilized for NCT have been mentioned in previous reviews, there were no conceptual updates on novel strategies of optimized NCT.

The review would provide updated insights into the novel strategies of 1) achieving highly effective nanocatalytic reactions, including the modification of nanocatalysts and TME‐modulating approaches. 2) Furthermore, the design and formulation of multifunctional nanozyme‐based systems which achieved targeted, synergistic therapy, and theranostic applications would be analyzed and concluded. Concise and concentrated comments and outlooks are illuminated at the end to outline the perspectives and the remaining challenges for next‐step strategies for further biomedical translation of NCT.

## 2 Development of the Highly Effective Nanocatalytic Reaction

### 2.1 The Innovation and Modification of High‐Performance Nanocatalysts

Fe_3_O_4_ NPs were the first nanozymes explored to perform dual enzyme‐like activity both in vitro and in vivo in a pH‐dependent manner.^[^ ^20^ ^]^ They presented catalase‐like (CAT) activity decomposing H_2_O_2_ into nontoxic H_2_O and O_2_ under neutral pH conditions, while showing POD activity under acidic condition disproportionating H_2_O_2_ into extremely toxic ROS—•OH.^[^ ^21^ ^]^ Up to date, several nanozymes with parallel nanocatalytic performance of Fe_3_O_4_ NPs (Co^2+^, Cu^2+^, Mn^2+^, and MOFs) have been discovered, but there were still amounts of challenges in implementing nanocatalytic medicine toward clinical applications such as poor biocompatibility, biodegradability, and unsatisfied in vivo catalytic efficiency.^[^ ^22^ ^]^ Recently, with the deepening research of advanced nanozyme, more biocatalysts that break the shackles of traditional nanozymes have emerged, which were mainly represented by the surface modification, 2D morphological engineering, and the application of single‐atom catalysts (SACs) for improved biocompatibility and NCT effect against tumor.

#### 2.1.1 Surface Modification of the Nanocatalysts

**Figure 1:**
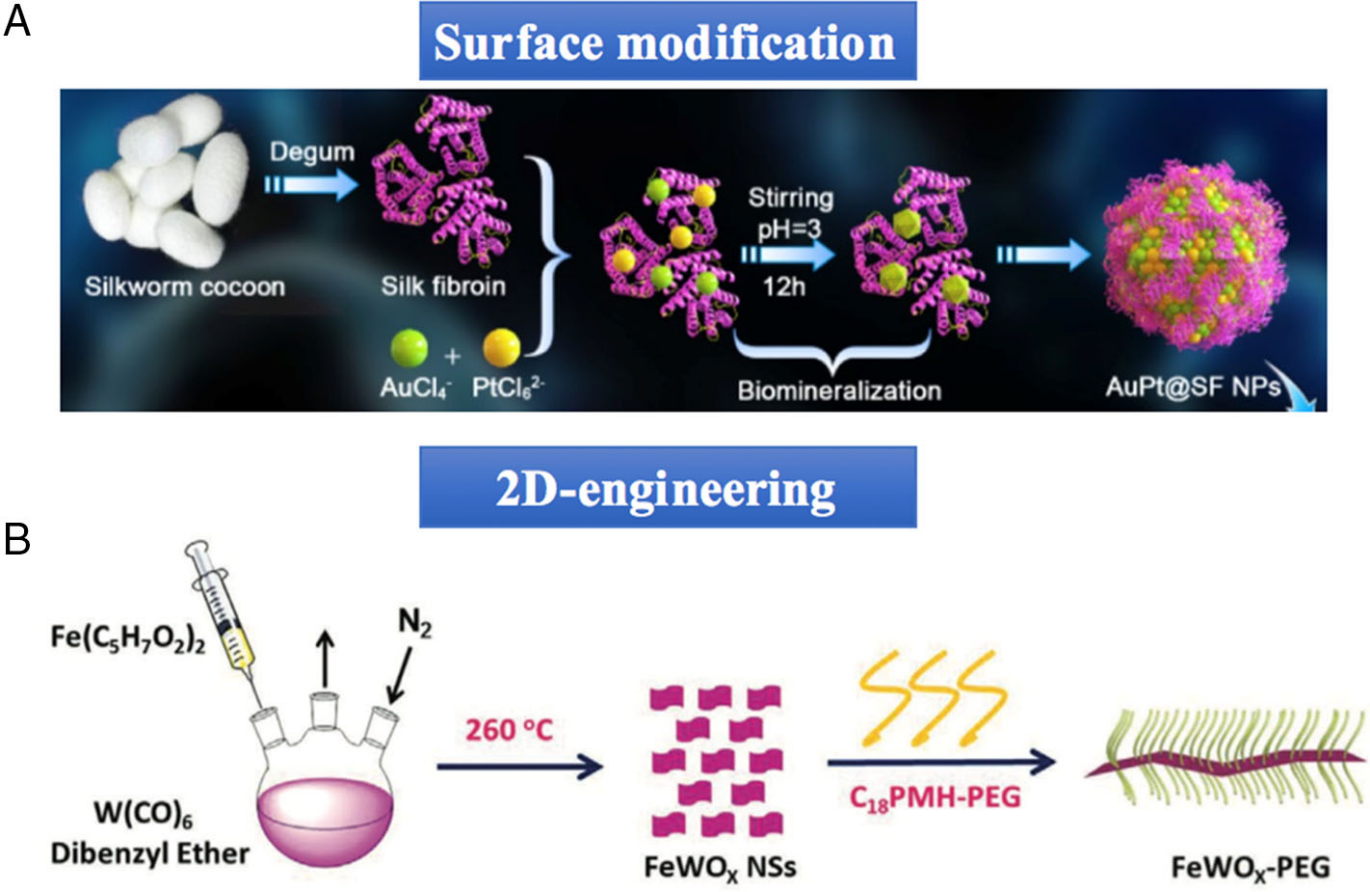
Surface modification and 2D engineering for enhanced nanocatalytic effect against cancer. A) The synthetic process of APS bimetallic nanozyme. Reproduced with permission.^[^ ^24^ ^]^ Copyright 2021, IVYSPRING. B) The synthetic process of FeWO_*X*_ nanosheets. Reproduced with permission.^[^ ^26^ ^]^ Copyright 2020, Wiley‐VCH.

Though quite a few nanomaterials were proved as efficient catalysts, their poor biodegradability and unclear biocompatibility made the great challenge for medical applications. As a result, the modification of the surface chemistry turned out to be the prerequisite for the in vivo experiments.^[^ ^23^ ^]^ Yang et al.^[^ ^24^ ^]^ reported that silk fibroin (SF) with abundant surface area which exhibited excellent aqueous stability could be applied as the mineralization inducer and sacrificial template to synthesize bimetallic nanozyme AuPt@SF (APS) through one‐step reaction (**Figure** **1**A). The as‐designed APS presented desirable biocompatibility and tumor accumulation in vivo; more importantly, it provided exceptional catalytic stability by the coating of SF. The SF‐coated nanozyme could easily convert the adsorbed O_2_ and intratumoral H_2_O_2_ into •OH and superoxide radicals (•O^2−^), respectively, by mimicking the functions of POD and oxidase. The anticancer process of elevated ROS production and glucose consumption via catalytic reaction effectively inhibited tumor growth, achieving irreversible oxidative stress destruction and deleterious tumor starvation. As a result, the study demonstrated the SF‐modified bimetallic nanozyme with favorable biosafety could induce considerable inhibition rate on tumor growth both in vitro and in vivo.

#### 2.1.2 Catalytic Application of 2D Nanosheets

In recent years, 2D nanosheets have gained great interest in virtue of their extraordinary 2D‐structured physical and chemical superiority compared with the conventional 3D nanomaterials.^[^ ^25^ ^]^ Gong et al.^[^ ^26^ ^]^ lately synthesized novel FeWO_*X*_ nanosheets with highly efficient POD‐like property via a thermal decomposition method (Figure 1B). Owing to the 2D structure advantages in maximally exposed Fe atoms and the high distribution of oxygen vacancies (catalytic sites) on its surface, the FeWO_*X*_ nanosheets showed desirable catalytic activity in decomposing H_2_O_2_ into •OH. Therefore, the as‐synthesized 2D nanozyme was proved to accelerate the catalytic efficiency than previously reported 3D nanozymes.

#### 2.1.3 Design of Single‐Atom Nanocatalyst for Cancer Therapy

**Figure 2:**
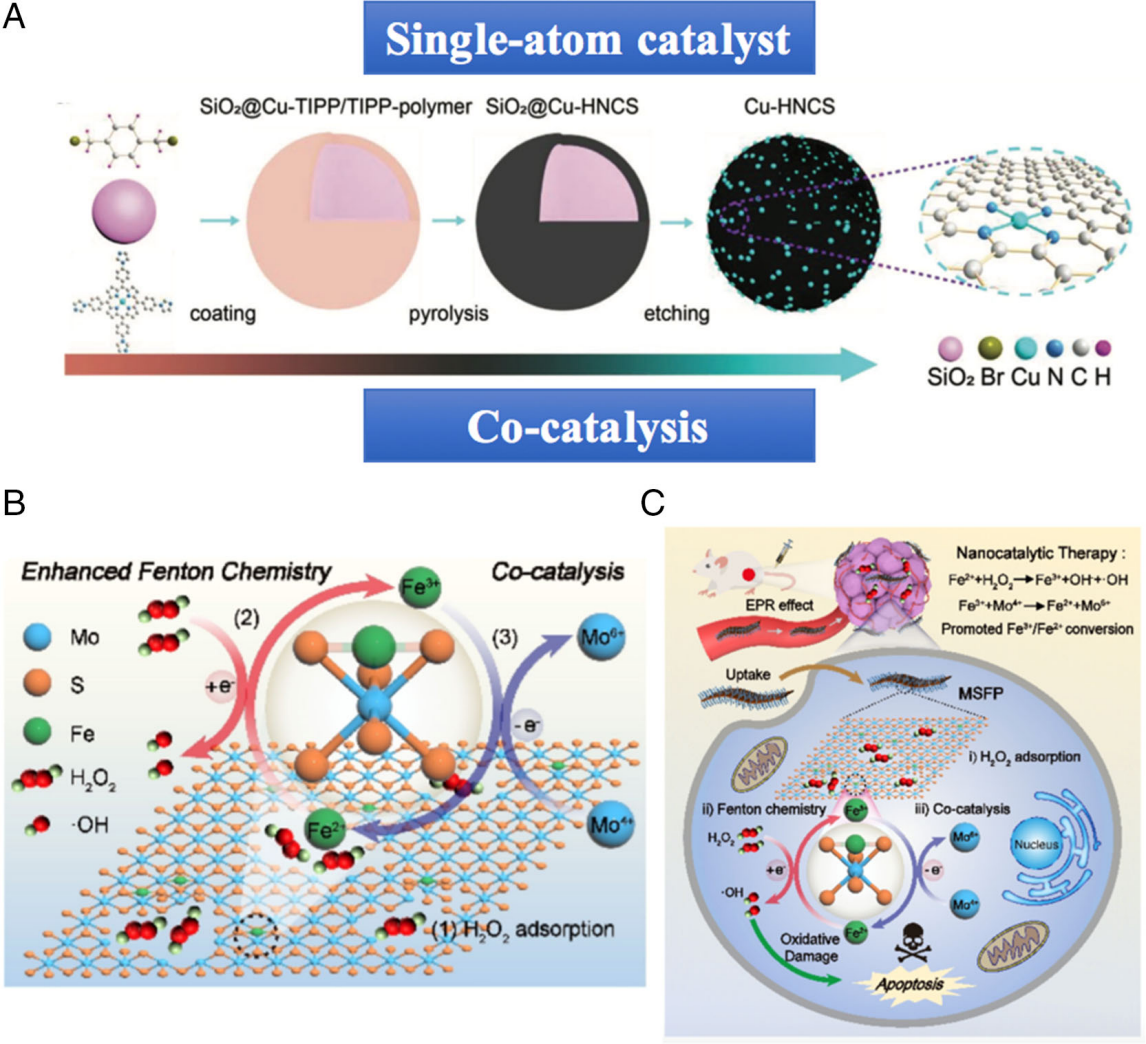
Design and construction of SACs and cocatalysis. A) The synthetic process of SACs (Cu‐HNCS) by organic coating, carbonization, and silica etching. Reproduced with permission.^[^ ^29^ ^]^ Copyright 2021, Wiley‐VCH. B) Chemical mechanism of cocatalysis induced single‐atom Fenton reaction. C) Schematic illustration of antitumor effect of the cocatalyst. Reproduced with permission.^[^ ^30^ ^]^ Copyright 2022, Wiley‐VCH.

SACs with preferable catalytic chemistry have been applied as nanozymes in the latest studies.^[^ ^27^ ^]^ SACs possessed excellent catalytic performance with the maximization of metal utilization, which could achieve desirable cancer suppression with relatively low metal concentrations.^[^ ^28^ ^]^ Inspired by the excellent properties of SACs as nanozyme, Lu et al.^[^ ^29^ ^]^ designed a hollow N‐doped carbon sphere doped with a single‐atom copper species (Cu‐HNCS) that could directly decompose both O_2_ and H_2_O_2_ to ROS without external stimuli, thus inducing an improved cancer‐inhibiting effect **(** **Figure** **2**A **)**. It should be noted that the turnover frequency of Fenton reaction by Cu‐HNCS was ≈5000 times higher than commercial Fe_3_O_4_ NPs. Moreover, the experimental outcomes on tumor‐bearing mice revealed that Cu‐HNCS nanozyme could significantly inhibit tumor growth and improve the survival rates. Importantly, the satisfying therapeutic outcomes realized by relatively low metal concentrations indicated the further biomedical application of SACs for cancer therapy.

Given that the efficacy of Fenton reactions suffered from the unsatisfactory Fe^3+^ to Fe^2+^ conversion kinetics, Yang et al.^[^ ^30^ ^]^ developed a cocatalytic concept in catalytic therapy by introducing a 2D MoS_2_ nanosheet atomically dispersed with Fe species. Taking the advantages of SACs and 2D nanosheets, Fenton reactions could be triggered by active sites of the single‐atom Fe species, while the abundant sulfur vacancies generated on the nanosheet facilitated electron capture by H_2_O_2_ for elevated •OH production **(**Figure 2B **)**. More importantly, under the support of the cocatalyst 2D MoS_2_, the conversion of Fe^3+^ to Fe^2+^ could be accelerated by the oxidation of active Mo^4+^ sites to Mo^6+^, thereafter improving the whole catalytic efficiency **(**Figure 2C **)**. In vitro and in vivo experiments exhibited a significantly enhanced anticancer effect of 2D conanocatalysts, implying great prospects in the strategy of 2D nanosheets and SACs for the enhancement of NCT.

### 2.2 Updated TME‐Modulating Approaches for the Enhancement of NCT

Experiments highlighted that Fenton catalysts gained better performance under acidic environment (pH 3.0–5.0), and the produced ROS were liable to be consumed by excessive GSH in the TME.^[^ ^31^ ^]^ In addition, the enzymatic performance of nanocatalytic reaction was influenced by intrinsic H_2_O_2_ as the catalytic substrate.^[^ ^32^ ^]^ As such, the modulation of the TME condition through increasing H_2_O_2_ levels, lowering pH, and reducing GSH concentration was the potential strategy to optimize the catalytic effect.

#### 2.2.1 Elevating the Levels of H_2_O_2_

Theoretically, the nanozyme could decompose the H_2_O_2_ into ROS continuously to induce necrosis and apoptosis of malignant cells; however, in practical terms, intracellular H_2_O_2_ level of tumor was not adequate to generate a high enough amount of •OH to trigger satisfactory catalytic effect against cancer.^[^ ^33^ ^]^ Previous studies have achieved elevated H_2_O_2_ levels to facilitate ROS generation by sequential chemical reactions of coloaded glucose oxidase (GOD) or Au NPs.[^32^, ^34^] Nevertheless, several issues remained to be resolved. First, glucose consumption was an oxygen‐dependent process; given the relatively hypoxic TME condition, the generation of H_2_O_2_ might be inhibited. In addition, lack of targeting modification would also enable the exogenous enzyme to react with glucose in normal tissues, reducing the selectivity of the reaction. As such, latest studies proposed updated strategies on elevating the levels of H_2_O_2_ by constructing novel nanocomposites without introducing additional substances.

**Figure 3:**
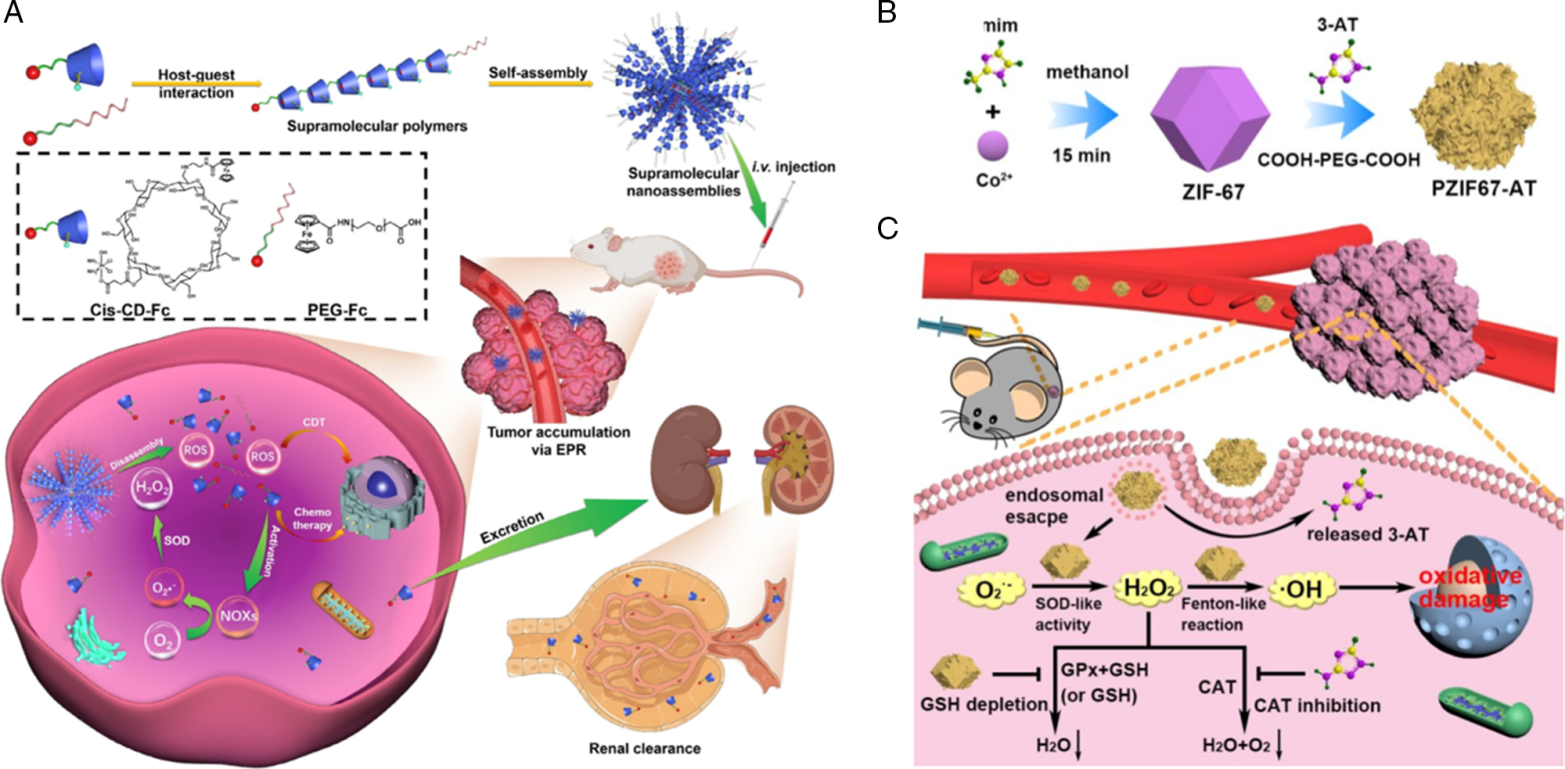
H_2_O_2_ elevating strategies to promote catalytic process for enhanced antitumor effect. A) Schematic illustration of the H_2_O_2_‐responsive supramolecular nanoassemblies for self‐augmented NCT. Reproduced with permission.^[^ ^35^ ^]^ Copyright 2021, Wiley‐VCH. B) Schematic illustration of the synthesis of PZIF67‐AT and C) the augmented antitumor mechanism and process of PZIF67‐AT. Reproduced with permission.^[^ ^36^ ^]^ Copyright 2020, American Chemical Society.

Yang et al.^[^ ^35^ ^]^ fabricated supramolecular NPs utilizing platinum (IV) complex‐modified β‐cyclodextrin–ferrocene conjugates as supramolecular monomers via a facile one‐step self‐assembly process **(** **Figure** **3**A **)**. The supramolecular NPs could dissociate efficiently under the exposure of H_2_O_2_ in TME and thereafter generate platinum (IV) prodrugs and •OH in situ. Intriguingly, the level of H_2_O_2_ would be significantly elevated when the platinum (IV) was reduced into cisplatin in the tumor tissue. As such, the as‐described supramolecular nanozyme was expected to overcome the limitation of the exogenous enzyme‐enabled cascade reaction and drug release. In addition, metabolic assays proved that the dissociated supramolecular NPs excreted via renal clearance, which may be desirable for long‐term biocompatibility of the nanocomposites.

Inspired by the metabolic balance of H_2_O_2_ in TME, Sang et al.^[^ ^36^ ^]^ designed and constructed a nanozyme‐based homeostasis disruptor of H_2_O_2_ for enhanced NCT **(**Figure 3B **)**. With highly SOD‐like and CAT‐inhibiting activities, the disruptor could accelerate the generation and restrict the elimination of H_2_O_2_ by interfering with H_2_O_2_ homeostasis, inducing elevated levels of H_2_O_2_ in TME **(**Figure 3C **)**. As a result, a Fenton‐like reaction could be further triggered and produce more harmful •OH to induce intracellular oxidative stress for enhanced NCT.

#### 2.2.2 Modulating the Levels of pH and GSH to Maximize the Catalytic Activity

**Figure 4:**
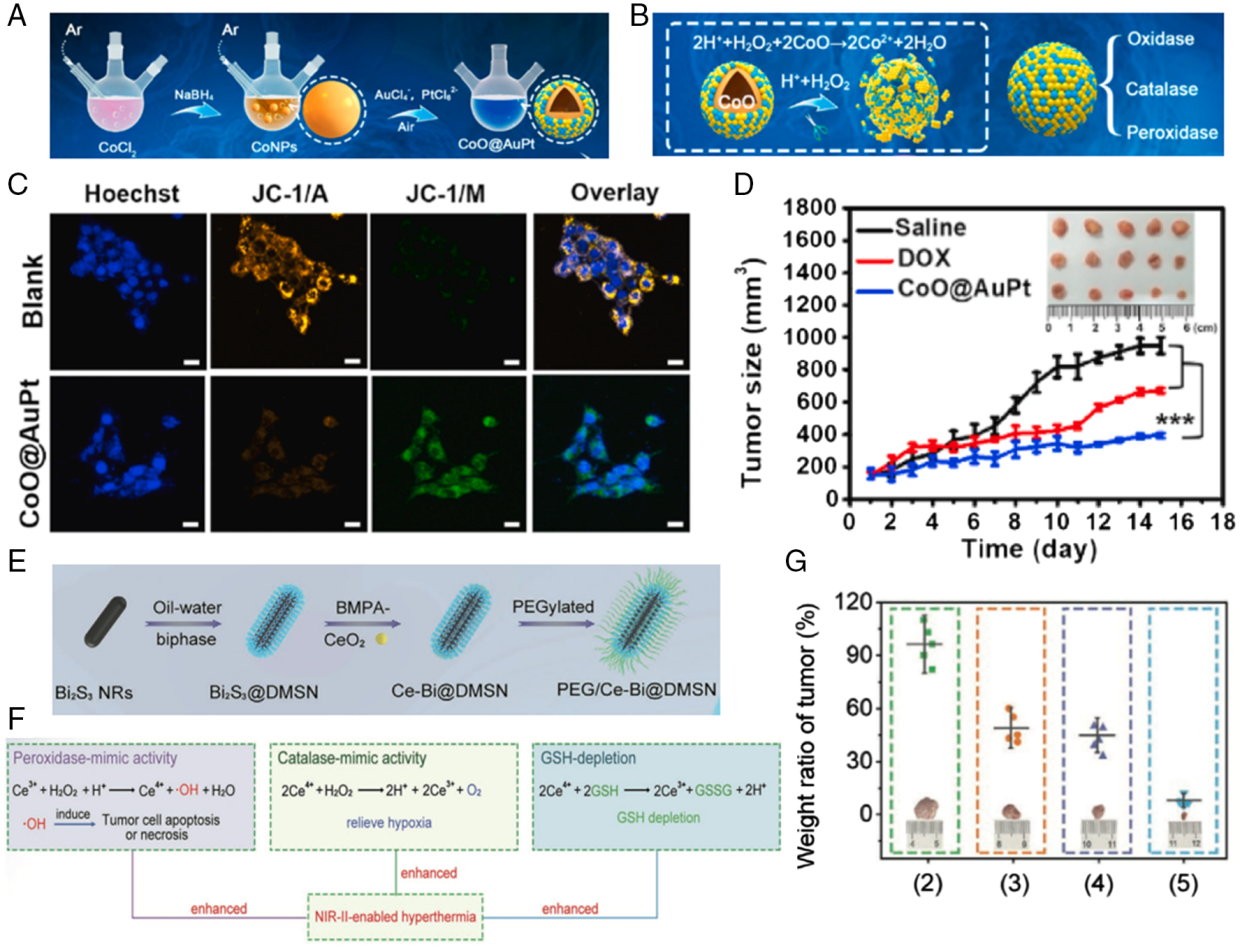
Enhanced nanocatalytic anticancer effects by modulating pH and GSH levels. A,B) Schematic illustration of the synthetic procedure of hollow CoO@AuPt NPs and its catalytic mechanisms. C) Mitochondrial damage of 4T1 cells following 8 h treatment with CoO@AuPt NPs measured by JC‐1 assay kit. JC‐1/M and JC‐1/A indicated the monomer and aggregated forms of JC‐1, respectively. D) Tumor growth curve of 4T1 tumor‐bearing BALB/c mice after receiving saline, DOX, or CoO@AuPt (inset: photos of dissected tumors on 15‐day treatment). Reproduced with permission.^[^ ^37^ ^]^ Copyright 2020, Elsevier Ltd. E) Schematic illustration of the successive synthetic procedures of the PEG/Ce‐Bi@DMSN nanozymes. F) Schematic illustration of dual enzyme‐mimic catalytic activities and GSH depletion. G) Proportion of tumor weight in various groups relative to that in untreated mice obtained after 20 days of treatment. 1) control (saline), 2) 1064 nm laser, 3) PEG/Bi_2_S_3_@DMSN + 1064 nm laser, 4) PEG/Ce‐Bi@DMSN, and 5) PEG/Ce‐Bi@DMSN + 1064 nm laser. Reproduced with permission.^[^ ^40^ ^]^ Copyright 2020, Wiley‐VCH.

To further maximize the catalytic activity by regulating the levels of pH and GSH, Fu et al.^[^ ^37^ ^]^ established a novel nanosystem based on hollow CoO@AuPt nanozyme (**Figure** **4**A). Utilizing Co NPs as sacrificial templates, the as‐synthesized CoO@AuPt was expected to protect the tiny Au/Pt nanosatellites from exposure and gain excellent stability during long‐term circulation. In addition, the penetration depth could increase in solid tumors once the structure of CoO@AuPt was disintegrated by acid environment. Free Co^2+^ could trigger the generation of ROS in the presence of HCO_3_^−^/H_2_O_2_ as a highly efficient Fenton‐like agent, and the disintegrated Au/Pt nanosatellites thereafter promoted multiple enzymatic activities. The TME regulation of the nanosystem was mainly reflected in the following aspects. First, Au/Pt nanosatellites could convert GSH into GSH disulde (GSSG), which notably decreased ROS consumption by GSH. Second, an increased amount of intracellular H_2_O_2_ was decomposed into •OH and O_2_ via POD‐ and CAT‐like activities by the nanocomposites, respectively. Third, the intrigue GOD‐mimic activity of Au/Pt nanosatellites could consume the intratumoral glucose in the presence of O_2_ and produce an elevated level of H_2_O_2_, which in turn promoted the generation of ROS species (Figure 4B). The highly efficient anticancer effect of the NCT‐based hollow CoO@AuPt NPs was confirmed both in vitro and in animal studies (Figure 4C,D), building up novel and promising therapeutic mechanisms by regulating the unique TME conditions.

CeO_2_ was recently acquired close attention in the field of nanocatalytic reactions owing to its unique mixed‐valence states of Ce^3+^ and Ce^4+^ and oxygen vacancies.^[^ ^38^ ^]^ Compared with natural enzymes, CeO_2_ could exhibit a Ce^3+^‐dependent enzymatic activity as POD over a wide range of temperature and pH.^[^ ^39^ ^]^ Consequently, regulating the Ce^3+^/Ce^4+^ ratio and obtaining more oxygen vacancies could maximize the POD‐mimic activity of CeO_2_. Moreover, CeO_2_ nanozymes could also perform CAT‐like activities due to their special properties in reversibly switching from Ce^4+^ to Ce^3+^ in mildly acidic environment. Given the as‐described distinctive characteristics of CeO_2_, Dong et al.^[^ ^40^ ^]^ reported the establishment of a nanocomposite called PEG/Ce‐Bi@DMSN **(**Figure 4E **)**, which was demonstrated to exhibit POD‐ and CAT‐mimicking activity, in addition to GSH depletion ability under TME **(**Figure 4F **)**. In detail, the ultrasmall CeO_2_ in the nanocomposite functioned as a highly efficient Fenton‐like agent to generate toxic ·OH in specific acidic TME status. Meanwhile, the nanozymes could destroy the antioxidant defenses of tumor so as to increase its vulnerability to ROS. Notably, Bi_2_S_3_‐based photothermal (PTT) effect under laser irradiation significantly strengthened the above‐described catalytic activities, and in vitro and in vivo experiments achieved an enhanced tumor suppression by the nanocomposite (Figure 4G).

## 3 Development of Multifunctional Nanozyme‐Based Systems

### 3.1 Targeted Nanosystems of NCT

Previous nanodeliveries of NCT were mainly based on the passive enhanced permeability and retention (EPR) effect of nanozyme, which inevitably caused undesirable tumor aggregation and side effects in normal tissues. Recently, a growing number of researchers have designed tumor‐specific targeting delivery nanosystems according to tumor biological characteristics, utilizing cellular and subcellular targeting receptors. The targeting engineering of nanozyme not only increased tumor selectivity and local aggregation, but also reduced side effects on normal tissues, which was of great importance for further biomedical translation.

#### 3.1.1 Targeting Effect of Extracellular Vesicles (EVs)

**Figure 5:**
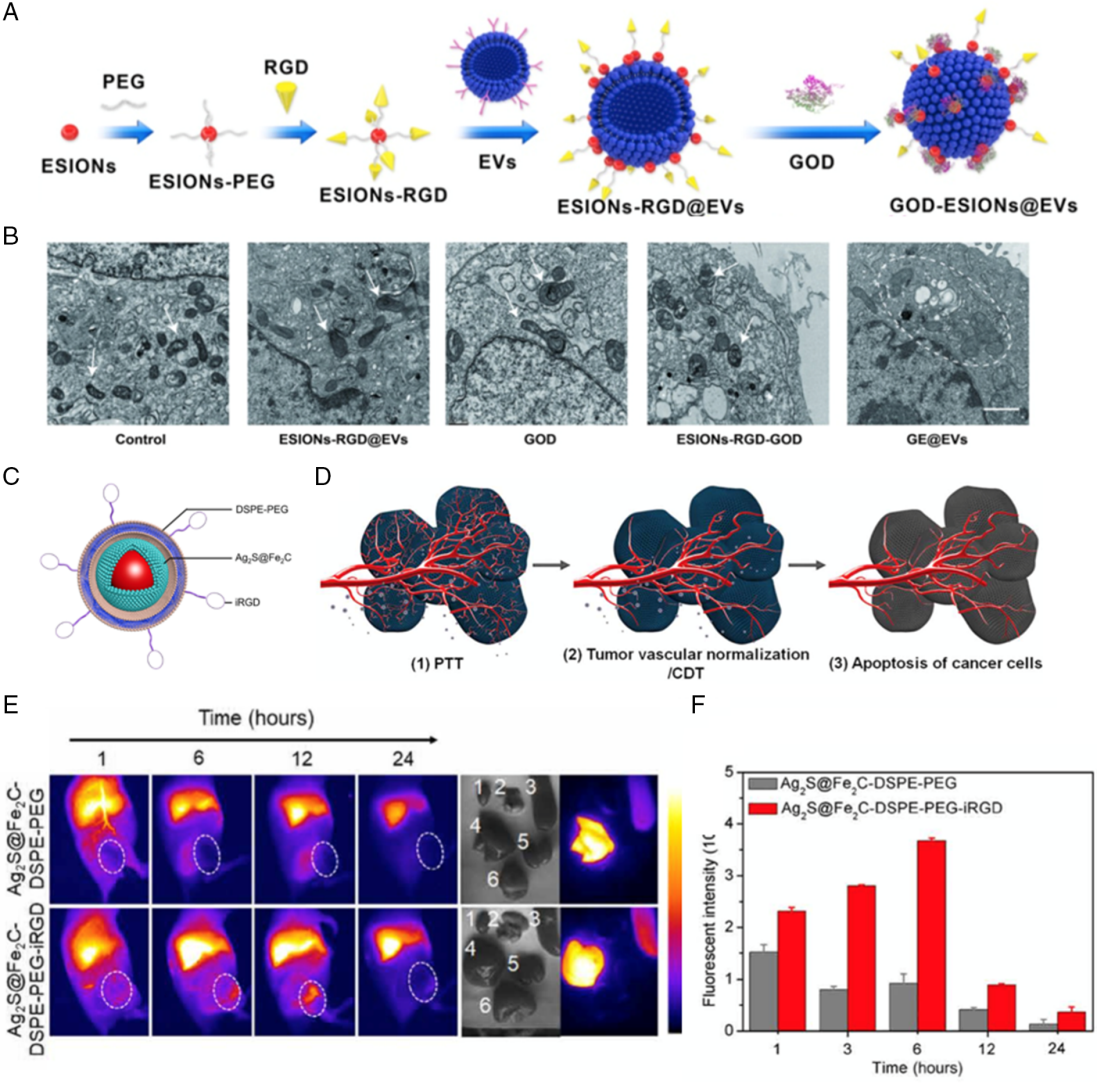
Targeted engineering of nanozyme‐based systems to facilitate enhanced NCT. A) Schematic diagram for the fabrication of GE@EVs. B) Bio‐TEM images of intracellular organelles after different treatments. The white arrow indicated the normal cell shape, and white dotted line circle indicated the swollen and deformed mitochondria and golgi bodies. Reproduced with permission.^[^ ^41^ ^]^ Copyright 2021, IVYSPRING. C) Schematic illustration of the designed Ag_2_S@Fe_2_C‐DSPE‐PEG‐iRGD core–shell heterojunctions. D) Schematic illustration of targeted NCT with tumor vascular normalization. E) Real‐time NIR‐II fluorescence images of 4T1 breast cancer‐bearing mice after intravenous injection of Ag_2_S@Fe_2_C‐DSPE‐PEG/‐iRGD. Ex vivo fluorescence images of heart (1), kidney (2), spleen (3), liver (4), lung (5), and tumor (6) (48 h after injection). F) The fluorescence intensities of the tumor after intravenous injection of Ag_2_S@Fe_2_C‐DSPE‐PEG/‐iRGD. Reproduced with permission.^[^ ^43^ ^]^ Copyright 2020, the American Association for the Advancement of Science.

Our work^[^ ^41^ ^]^ recently discovered EVs collected from hepatocellular carcinoma (HCC) cells as an effective targeted carrier for NCT. As natural lipid bilayer membranes secreted by living cells, EVs were endowed with optimal biocompatibility and degradability.^[^ ^42^ ^]^ In addition, EVs as a nanocarrier were expected to promote the intracellular endocytosis of EV–membrane fusion and facilitate targeted therapy via unique membrane‐targeting capability. The work illustrated that EVs‐loaded GOD could serve as the initiating enzyme to produce H_2_O_2_ by catalyzing the intracellular glucose **(** **Figure** **5**A **)**. The downstream ultrasmall iron oxide NPs (ESIONs) thus transformed the excessive H_2_O_2_ to ·OH via Fenton‐like reaction within the HCC region, triggering mitochondria damage of HCC cells **(**Figure 5B **)**. Especially, the active targeting capability of EVs could achieve more efficient NCT by gathering increased amounts of sequential nanocatalysts into the HCC region. The targeted performance of EV‐engineered nanozyme presented highly efficient HCC inhibition in vitro and in vivo, broadening versatile bioapplications of EVs as a promising carrier for NCT.

#### 3.1.2 Targeting Effect of Tumor‐Homing Penetration and Tumor Vascular Normalization

To formulate nanozyme that precisely produces ROS within tumor tissue and decreases the off‐target destruction to adjacent normal tissues, Wang et al.^[^ ^43^ ^]^ reported a “TME‐unlocking” paradigm through the combination between targeting‐engineered nanozyme and tumor vascular normalization to combat tumor growth **(**Figure 5D **)**. They first synthesized monodispersed core–shell Ag_2_S@Fe_2_C heterogeneous NPs **(**Figure 5C **)**. Next, to achieve tumor‐targeting effects, a nanosystem (Ag_2_S@Fe_2_C‐DSPE‐PEG‐iRGD) was established by surface conjugation of a tumor‐homing penetration peptide‐modified distearoyl phosphoethanolamine‐PEG‐iRGD peptide (DSPE‐PEG‐iRGD). The iRGD**‐**modified nanozyme exhibited better targeting performance in vivo **(**Figure 5E,F **).** Notably, a more favorable anticancer effect was observed in 4T1 breast cancer‐bearing mice by the targeted NCT combined with the bevacizumab‐based tumor vascular normalization.

### 3.2 Stimuli–Responsive Nanosystems for Enhanced Antitumor Effect

Several nanomaterials with distinct catalytic functions possessed great response to laser/ultrasound (US)/magnetism at the same time, such as the PTT and photodynamic (PDT) process.^[^ ^44^ ^]^ Previous studies have proved the combinational effect of NCT and PTT/PDT triggered by laser irradiation. Nevertheless, the stimuli‐responsive process and antitumor effect were not well illustrated. This part would take the representative recent studies to illustrate the enhanced antitumor effect of NCT under various external stimulus.

#### 3.2.1 Photoresponsive NCT

**Figure 6:**
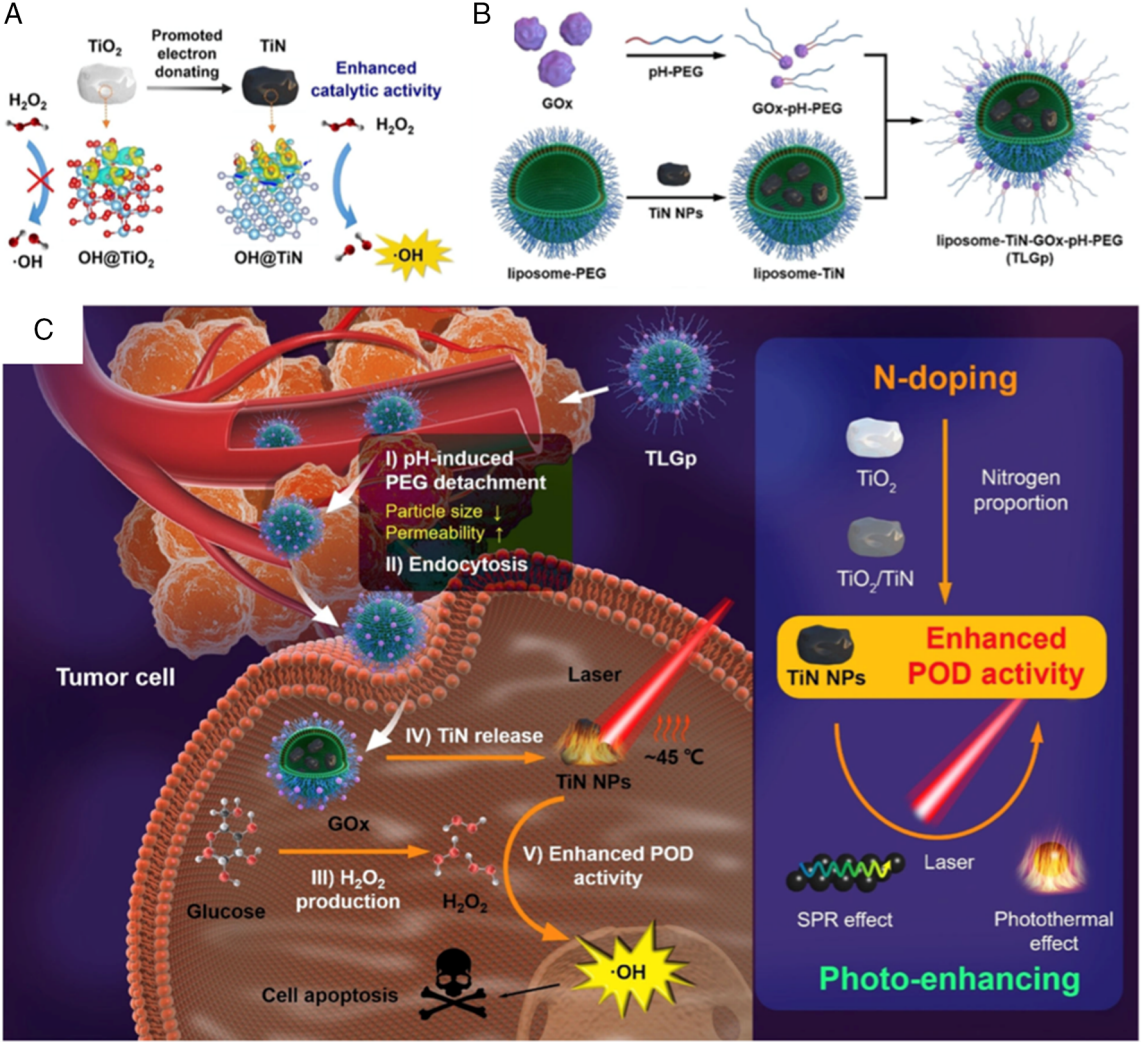
Schematic illustration of the construction and catalytic process of cascade TLGp. A) Effect of nitrogen doping in TiO_2_ NPs on their POD activity. B) Synthetic process of TLGp nanosystem. C) The factors affecting the POD activity of Ti‐based nanozymes and pH‐responsive/mild PTT‐mediated cascade NCT of TLGp nanosystem. Reproduced with permission.^[^ ^45^ ^]^ Copyright 2021, Wiley‐VCH.

To explore the impact of PTT effect on NCT, Liu et al.^[^ ^45^ ^]^ investigated the contributing factors of catalytic performance by surface engineering of nonmetallic atom doping and laser irradiation **(** **Figure** **6**A,B **)**. As the experiment indicated, POD reactivity of the Ti‐based nanozyme was significantly enhanced via nitrogen doping (TiN). In addition, owing to the excellent PTT effect of TiN induced by near‐infrared (NIR) adsorption, its enzymatic performance could be further improved by laser irradiation **(**Figure 6C **)**. As a result, they successfully synthesized a TME‐ and laser‐responsive nanozyme: TiN‐coated liposomes connecting PEG‐modified GOD with pH‐responsive manners. The integration of coloading GOD to achieve self‐supply of H_2_O_2_, nitrogen doping, and the photoresponsive property of TiN NPs enabled a satisfied tumor inhibition with minimal side effects in vivo.

#### 3.2.2 US‐Triggered NCT

**Figure 7:**
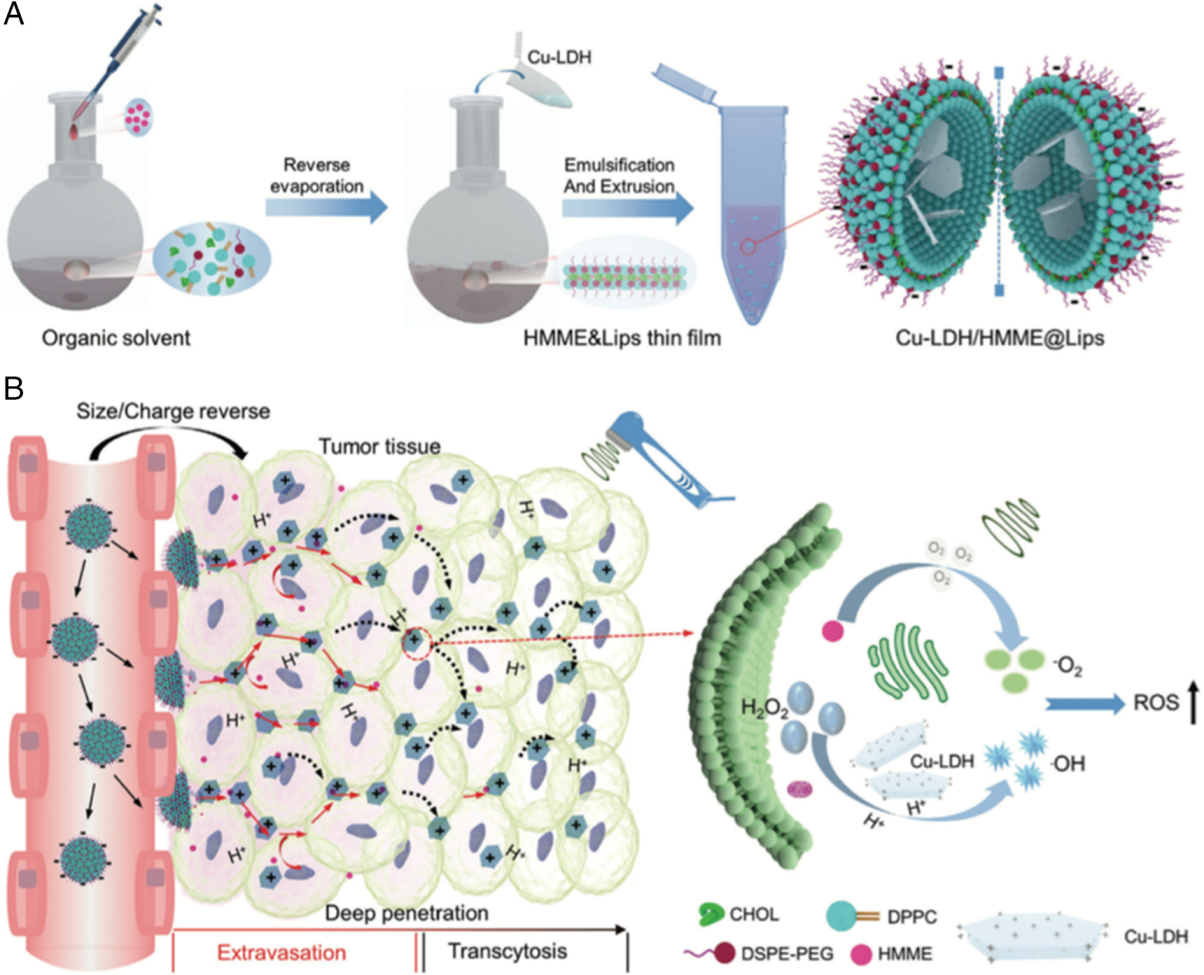
A) Schematic illustration of the synthesis of Cu‐LDH/HMME@Lips. B) Schematic illustration of the intracellular antitumor effect of dual‐size/charge‐switchable Cu‐LDH/HMME@Lips that transported in poorly permeable solid tumor models and the mechanism of ROS generations under US. Reproduced under the terms of the CC‐BY 4.0 license.^[^ ^46^ ^]^ Copyright 2021, The Authors, published by Wiley‐VCH.

Though various approaches have been attempted to increase the accumulation of nanomedicine at tumor site, it still remained a great challenge in animal experiments considering the largely blocked intratumoral infiltration. Wu et al.^[^ ^46^ ^]^ designed an US‐triggered dual‐size/charge‐switchable nanozyme (labled as Cu‐LDH/HMME@Lips) **(** **Figure** **7**A **)**, which was expected to break the limitations for the treatment of deep solid tumors. Liposomes acted as a carrier to codeliver enzymatic copper‐doped layered double hydroxide (Cu‐LDH) and HMME, a sonosensitizer. The surface advantages in large area and negative electricity endowed the nanozyme with long‐period blood circulation for tumor accumulation. After entering tumor tissues, it could rapidly disassemble themselves to release the size‐reduced Cu‐LDH nanosheets upon US irradiation; simultaneously, the dissembled sonosensitizer HMME could mediate the generation of ^1^O_2_ species **(**Figure 7B **)**. Consequently, upon US irradiation, the Cu‐LDH nanozyme was expected to infiltrate deeply into the tumor tissue, thus producing highly toxic •OH by the NCT process, and the associated generation of ^1^O_2_ by HMME could achieve a synergistic ROS effect, significantly suppressing the tumor growth in vivo. Such promising outcomes provided a prospect on the US‐responsive nanozyme to improve NCT efficacy against solid tumors.

#### 3.2.3 Magnetic‐Triggered NCT

**Figure 8:**
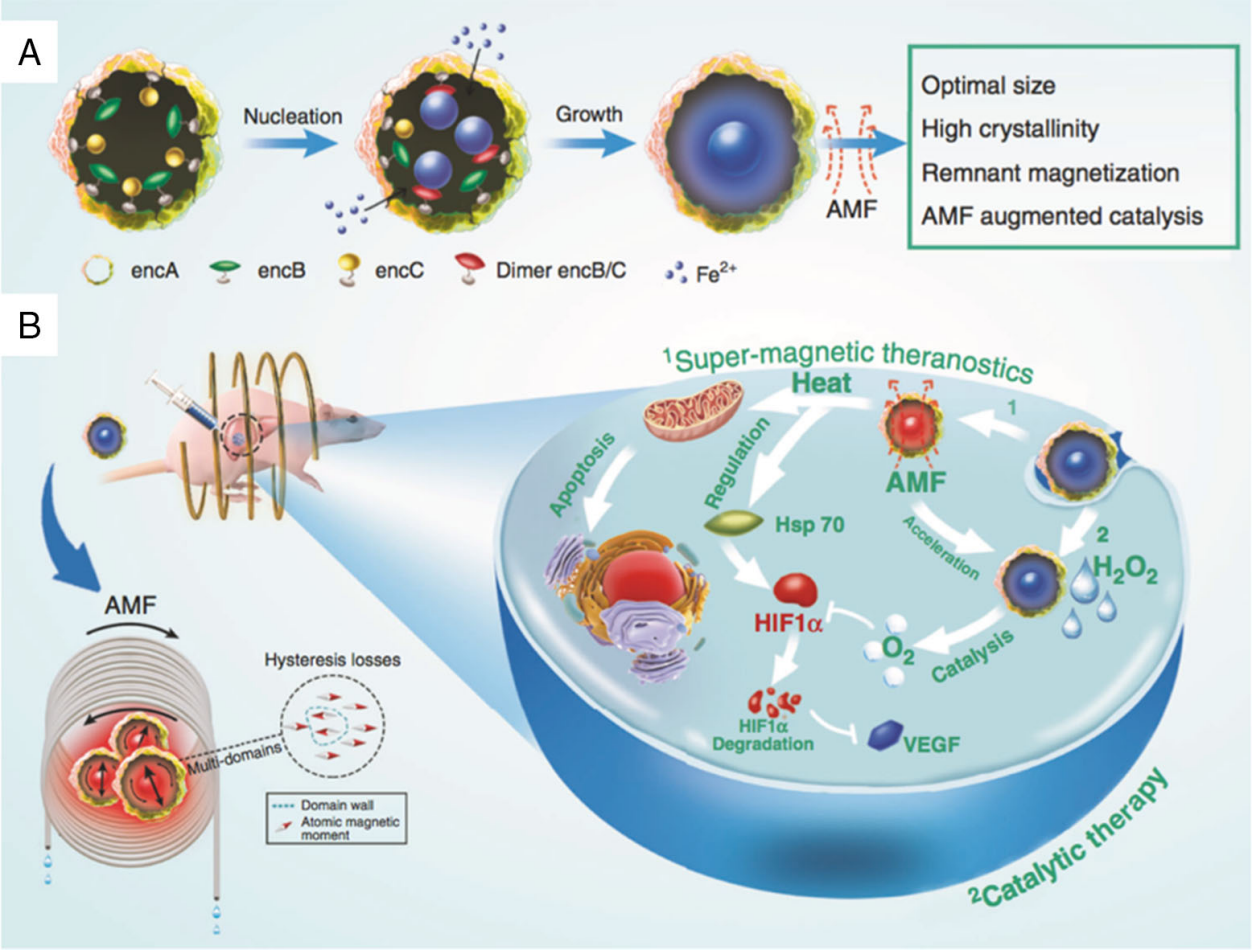
Schematic illustration of the magnetic‐triggered NCT. A) Schematic of an encABC and its biomimetic mineralized procedure. B) eMIONs accumulated in tumor sites and suppressed tumor growth effectively. The retaining of eMIONs in the tumor region promoted O_2_ production, decreased the expression of HIF‐1α, and VEGF. Afterward, magnetic hyperthermia and improved the catalytic efficacy could be achieved under alternative magnetic field, represented as magnetocatalytic therapy. Reproduced under the terms of the CC‐BY 4.0 license.^[^ ^48^ ^]^ Copyright 2020, The Authors, published by Springer Nature.

Magnetic hyperthermia therapy (MHT) was a novel minimally invasive management for cancer which has been applied in clinical practice.^[^ ^47^ ^]^ Among the various agents of MHT, iron oxide nanocomposites (IONs) were under widespread application due to their biocompatibility and unique magnetic property. Endowed with excellent capability as an MHT agent and nanozyme, studies have investigated the possibility of magnetic‐enhanced NCT based on IONs. Zhang et al.^[^ ^48^ ^]^ developed a facile and efficient strategy via a green biomineralization procedure for the synthesis of encapsulin‐produced magnetic IONs (eMIONs) **(** **Figure** **8**A **)**. With ≈100% crystallinity of Fe_3_O_4_, eMIONs were featured with preferable magnetic saturation and significantly improved the energy dissipation rate to promote the magnetic‐to‐thermal conversion capacity. More importantly, eMIONs presented as intrinsic CAT‐like nanozymes and achieved improved enzymatic performance under alternative magnetic field **(**Figure 8B **)**. The magnetic‐responsive magnetocatalytic synergistic therapy showed remarkable suppression on tumor growth of orthotopic HCC‐bearing mice and almost doubled their survival time.

### 3.3 Multifunctional Nanosystems for Synergistic Cancer Therapy

Nanosystems with synergistic therapeutic capabilities have been under extensive investigation lately, where it was highly desirable to design multifunctional nanosystems with the integration of multitherapeutic agents into one nanoplatform.^[^ ^49^ ^]^ Several studies have integrated chemodrugs into nanozyme‐based systems to achieve synergistic therapy with improved antitumor effect. With the development of tumor molecular biology, the targeted, immune, and gene‐editing drugs with excellent tumor selectivity have emerged and broken the limitations of inherent nonspecificity and side effects of chemotherapy. Recent frontier researches have also focused on the synergistic NCT and molecular tumor therapy by developing multifunctional nanosystems.

#### 3.3.1 Synergistic Gene Editing and Catalytic Therapy

**Figure 9:**
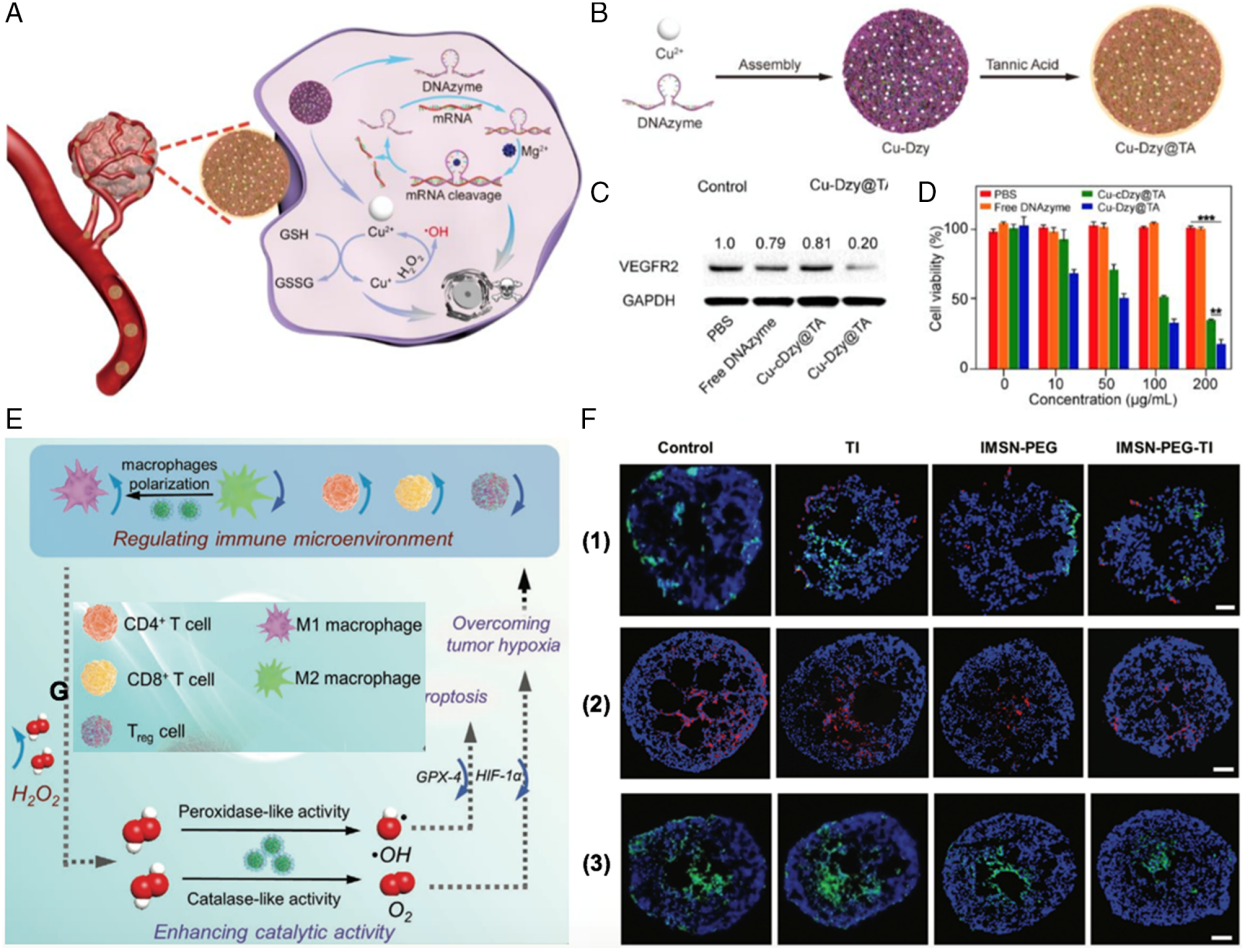
Construction of nanozyme‐based nanosystems for synergistic therapy. A,B) Schematic illustration of the synthesis of Cu‐Dzy@TA and the use of Cu‐Dzy@TA for dual‐catalytic tumor therapy. C) Western blot analysis of VEGFR2 protein in 4T1 cells after different treatments. D) Viability of 4T1 cells after incubating with different samples for 24 h. Reproduced with permission. Copyright 2021, Wiley‐VCH.^[^ ^50^ ^]^ E) Schematic illustration of tumor therapy for IMSN‐PEG‐TI; (F1‐3) Immunofluorescence images of macrophages distribution in MCTS (red: M1 macrophages, green: M2 macrophages, blue: nuclei); HIF‐1α (red: HIF‐1α, blue: nuclei); and GPX4 (green: GPX4, blue: nuclei). Reproduced with permission. Copyright 2020, Wiley‐VCH.^[^ ^53^ ^]^

Liu et al.^[^ ^50^ ^]^ developed a facile approach to synthesize hybrid nanostructures containing Cu^2+^ and DNAzyme with extremely high loading capacity **(** **Figure** **9**A,B **)**. The Cu‐DNAzyme nanosystem enabled favorable codelivery of Cu^2+^ and DNAzyme into tumor tissues for synergistic catalytic therapy. The disintegrated Cu^2+^ could be reduced to Cu^+^ by GSH and subsequently catalyzed intratumoral H_2_O_2_ to ·OH for the inhibition against cancer; meanwhile, the 10‐23 DNAzyme induced the catalytic cleavage of VEGFR2 mRNA and activated gene silencing for gene therapy **(**Figure 9C,D **)**. The as‐described nanosystem Cu‐Dzy@TA was demonstrated to improve the local accumulation of Cu^2+^ and DNAzyme, thus achieving excellent anticancer outcomes by dual‐enzyme‐induced catalytic therapy in vivo (including intracellular GSH depletion, Cu^+^‐based Fenton‐like reaction, and DNAzyme‐triggered gene silencing).

#### 3.3.2 Synergistic Immune and Catalytic Therapy

Though the predominance of NCT against cancer has been revealed by recent studies, its therapeutic outcomes were still impeded by several factors of TME, such as immunosuppressive and hypoxia microenvironment,^[^ ^51^ ^]^ which provided the great possibility of synergistic NCT and immunotherapy. It was reported that tumor‐associated macrophage (TAM) could be influenced by TME. Frequently, TAM expressed M2 phenotype, and it presented immunosuppressive activities. In contrast, the polarization to M1 macrophage could relieve the immunosuppression so as to increase intratumoral H_2_O_2_ level to potentially strengthen catalytic performance of nanozymes.^[^ ^52^ ^]^ As such, Xu et al.^[^ ^53^ ^]^ designed an immunomodulation‐enhanced strategy of NCT, which for the first time attained the synergism between TME regulation and nanozyme. The strategy was defined as the formulation of a H_2_O_2_‐responsive iron manganese silicate NP (IMSN) nanozyme and the subsequent loading of TGF‐β inhibitor (TI) into PEGylated IMSN (IMSN‐PEG‐TI) for Fe‐based catalytic therapy **(**Figure 9E **)**. IMSN with mixed‐valance conditions was proved to possess high POD‐ and CAT‐like performance under acidic environment. In particular, TI was loaded to promote polarization of macrophage from M2 to M1, which could effectively regulate TME through overcoming tumor hypoxia and increasing the ratio of M1 to M2 macrophages, CD4+/CD8+ T to Treg cells. As a result, the nanozyme combined with immunomodulation paradigm via IMSN‐PEG‐TI nanocomposites remarkably decreased the expression of glutathione peroxidase (GPX4) in cancer cells **(**Figure 9F **)**, showing favorable antitumor performance against CT26 tumor xenograft mice models with the tumor suppression rate of 87.5%.

**Figure 10:**
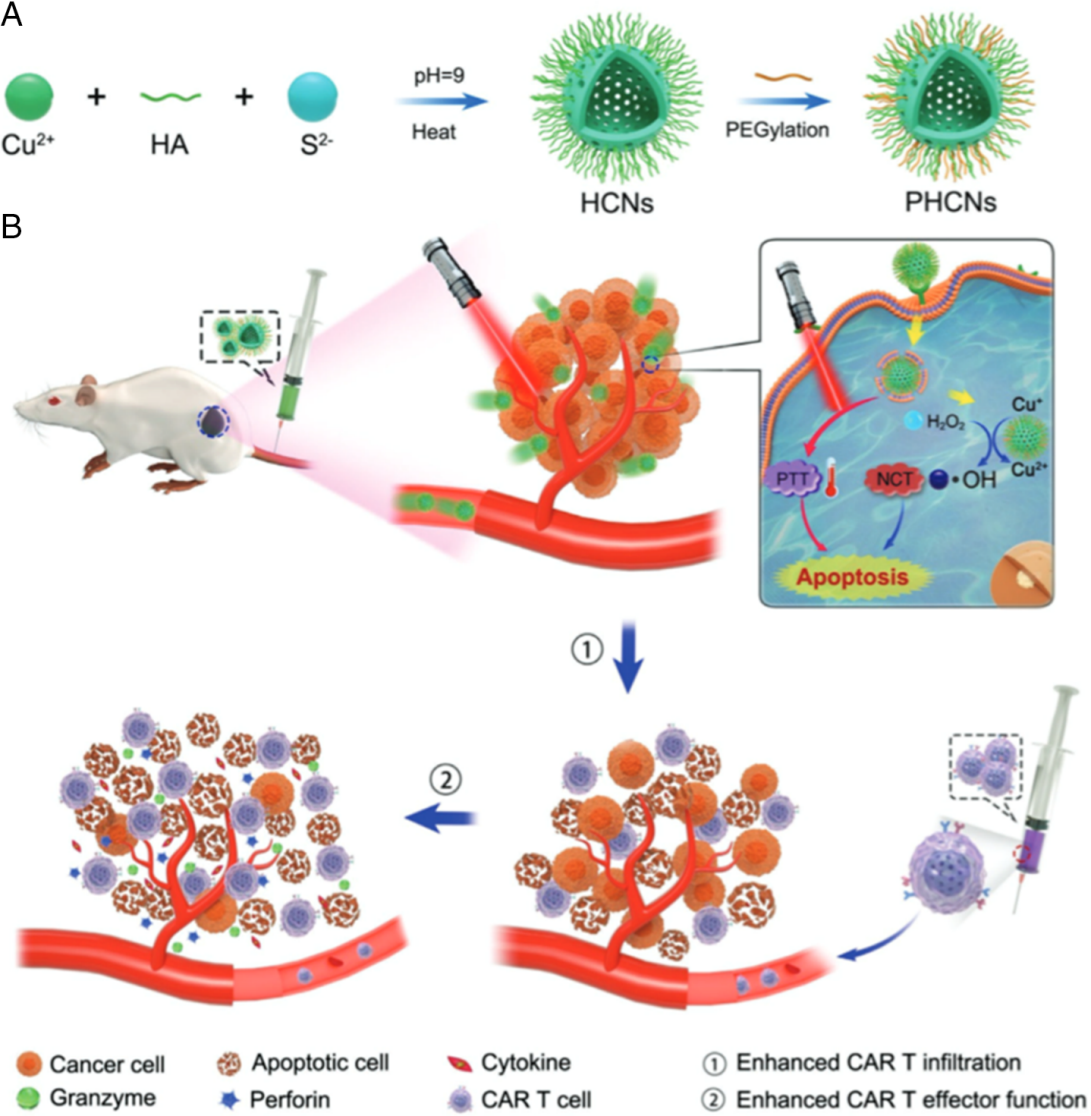
Schematic illustrations of the A) preparation procedure of PHCNs. (B) Nanozyme‐mediated PTT effect of enhanced infiltration and effector function of CAR‐T cells in solid tumors. Reproduced with permission.^[^ ^57^ ^]^ Copyright 2021, Wiley‐VCH.

Recently, chimeric antigen receptor (CAR) T‐cell therapy has aroused great potential for the therapy of non‐small cell lung cancer in preclinical studies.^[^ ^54^ ^]^ However, it remained a formidable challenge for suppressing solid tumors given the heterogeneous and immunosuppressive TME.^[^ ^55^ ^]^ Considering that nanozyme has been confirmed to exhibit advantages in regulating the immunosuppression of TME,^[^ ^56^ ^]^ Zhu et al.^[^ ^57^ ^]^ developed a nanocomposite (HA@Cu_2_‐X_S_‐PEG PHCN) achieving synergistic NCT and CAR‐T therapy to facilitate enhanced antitumor effect against solid tumors **(** **Figure** **10**A **)**. Experiments showed that nanozyme‐mediated PTT effect could disrupt the extracellular matrix and increase blood perfusion of tumor tissues, which promote the infiltration of CAR‐T cells. Meanwhile, ROS generated by NCT weakened tumor immune resistance and made it more vulnerable to CAR‐T cells. Furthermore, the release of tumor‐specific antigens by PTT process also triggered the recruitment and activation of antigen‐specific CAR‐T cells in solid tumors **(**Figure 10B **)**. The complementary superiorities of NCT and immunotherapy enabled them as promising synergistic candidates for enhanced efficiency against solid tumors.

### 3.4 Design of Multifunctional Theranostic Nanosystems

Multifunctional theranostic nanosystem integrating multimodal diagnostic and therapeutic agents to facilitate theranostic applications has inspired widespread interest in the past few years.^[^ ^58^ ^]^ Considering the process of NCT was hard to detect and evaluate in vivo, there is an urgent need to develop theranostic nanosystems to achieve real‐time monitoring of NCT. Several studies have attempted to enable the NCT with various types of diagnostic modes by the establishment of multifunctional theranostic nanosystems, which would further promote the clinical applications of NCT.

#### 3.4.1 NIR‐II Fluorescence‐Monitored NCT

**Figure 11:**
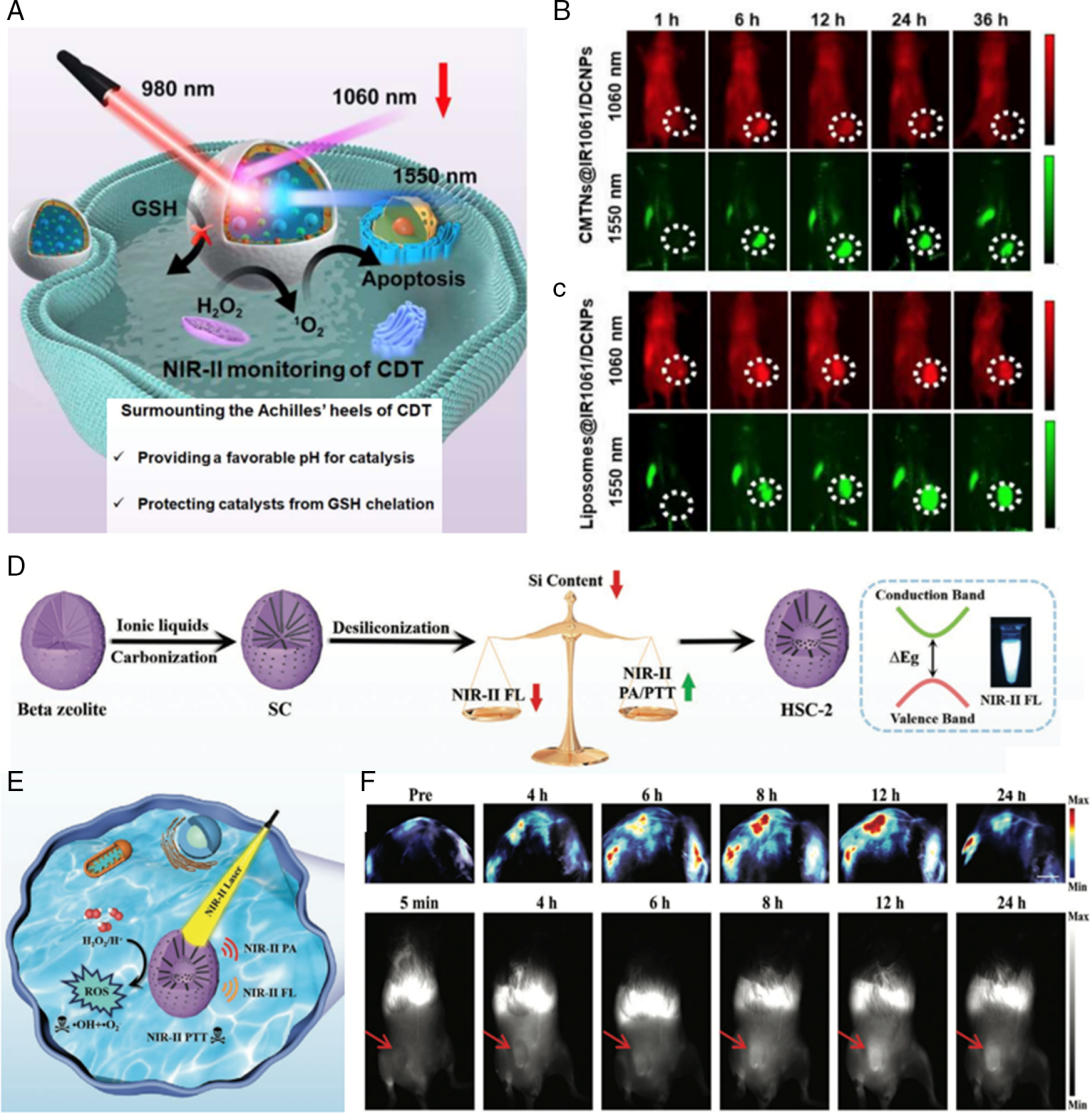
Establishment of theranostic nanosystems for real‐time monitoring of the antitumor effect of NCT. A) Schematic illustrating the application of CMTNs to efficiently generate ^1^O_2_ in dark hypoxia for NIR‐II ratiometric fluorescence‐monitored tumor NCT. B) In vivo NIR‐II fluorescence images of MCF‐7 tumor‐bearing mice after intravenous injection with CMTNs@IR1061/DCNPs or C) liposomes@IR1061/DCNPs. Reproduced with permission.^[^ ^59^ ^]^ Copyright 2021, Wiley‐VCH. D,E) Schematic illustration of the synthesis of HSC‐2 and adjustable PA/fluorescence imaging‐guided PTT/catalytic therapy in NIR‐II window. F) PA images of tumor in mice at different time points after intratumor injection of HSC‐2 under 808 nm laser (0.3 W cm^−2^) irradiation (1064 nm) and NIR‐II fluorescence images of 4T1 tumor‐bearing mice after systemic administration of HSC‐2 (1000 LP and 100 ms). Reproduced with permission.^[^ ^60^ ^]^ Copyright 2021, Wiley‐VCH.

To overcome the deficiency of nanozyme which warranted specific pH to maintain activity and became inactivated by GSH chelation, Chen et al.^[^ ^59^ ^]^ presented a catalytic microenvironment‐tailored nanoreactors (CMTN), formulated by surface conjugation of MoO_4_^2−^ catalyst and alkaline sodium carbonate (ACS). MoO_4_^2−^exhibited superior catalytic performance to yield ^1^O_2_ from H_2_O_2_ at pH 10.0–11.0. Given the impermeability of liposomal lipid membrane to ions and GSH, catalytic microenvironment‐tailored nanoreactors (CMNTs) involving confined MoO_4_^2−^ catalyst and ACS in the aqueous cavity of liposomes could provide an optimal catalytic pH to MoO_4_^2−^ and prevent GSH chelation‐induced catalyst inactivation **(** **Figure** **11**A **)**. Such a tailored TME associated with free diffusion across the liposomal membrane of reactant H_2_O_2_ and product ^1^O_2_, enabled the CMNTs with superior nanocatalytic efficacy. In addition, ROS‐sensitive IR1061 with NIR‐II fluorescence was incorporated into the CMTNs to real time monitor the generation of ^1^O_2_ catalyzed by MoO_4_^2−^. The as‐synthesized nanocomposites not only proposed a versatile strategy to reinforce the enzymatic performance by forming a TME‐tailored reaction, but also confirmed the great potential of NIR‐II fluorescence‐guided NCT for tumor theranostic in vivo **(**Figure 11B,C **)**.

#### 3.4.2 Multimodel Optical Imaging‐Monitored NCT

Diagnostic imaging in the NIR‐II biowindow has emerged as a novel strategy for highly precise tumor detection in latest researches. Zheng et al.^[^ ^60^ ^]^ lately developed a NIR‐II photoacoustic (PA)/NIR‐II fluorescence imaging‐monitored nanozyme (HSC‐2) to guide precise synergistic PTT‐catalytic antitumor therapy **(**Figure 11D,E **)**. Due to the adsorption capacity of ionic liquid, the electronic structure of the zeolite nano‐Beta could be turned from the indirect bandgap to direct bandgap via doping carbon in the framework, thus endowing it with remarkable performance in NIR‐II fluorescence emission. As the experiment indicated, HSC‐2 also possessed PTT and POD‐like capability under 1064 nm NIR irradiation, which could achieve a synergistic effect of ROS and hyperthermia, realizing satisfactory suppression on tumor growth. Especially, such a synergistic therapeutic process was guided by real‐time adjustable PA/fluorescence imaging in NIR‐II window **(**Figure 11F **)**. The novel cancer theranostic by formulating all‐in‐one nanozymes in this work would open up a new dimension in cancer management by expanding the applications of multimodal optical imaging in NIR‐II biowindow.

#### 3.4.3 3D Multispectral PA‐Monitored NCT

**Figure 12:**
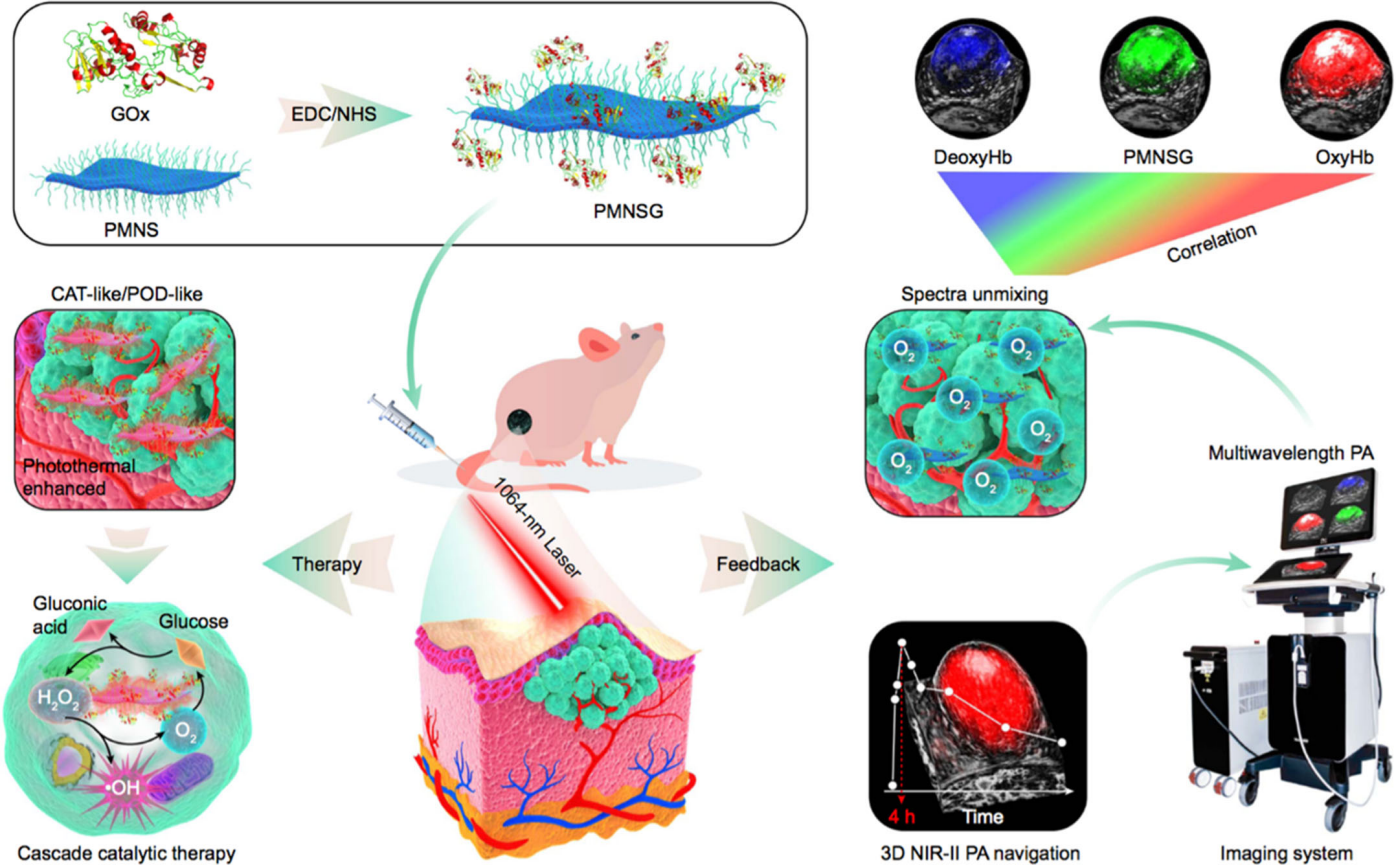
Schematic illustration of the preparation and application of cyclic cascade catalytic systems of PMNSG for NCT under the navigation of 3D PA imaging. Reproduced under the terms of the CC‐BY 4.0 license.^[^ ^61^ ^]^ Copyright 2022, The Authors, published by Springer Nature.

To achieve noninvasive visualization of dynamic molecular events of NCT in real time, Lei et al.^[^ ^61^ ^]^ formulated a dual enzyme‐driven cyclic reaction nanosytem which could apply 3D multispectral PA molecular imaging to monitor NCT in vivo. The nanosystem was composed of a 2D Pd‐based nanozyme connected with GOD, which could arouse the variation of the PA signal via endogenous molecules. Inspired by the nanozyme‐responsive PA imaging, the study mapped the 3D PA signals of dynamic endogenous and exogenous molecules associated with the catalytic process, thus achieving a real‐time noninvasive visualization of NCT **(** **Figure** **12** **)**. Consequently, the study presented the favorable prospect in imaging‐guided (3D multispectral PA imaging) theranostics especially in feedback‐looped cascade NCT.

## 4 Conclusion and Outlook

As Fe_3_O_4_ NPs were found to possess catalytic activity which could produce toxic ROS for NCT against cancer, numerous nanomaterials with unique catalytic activity have been developed, opening up a new chapter in tumor catalytic therapy.^[^ ^62^ ^]^ With the deepening insight into the NCT, the research focus has also extended from the discovery of high‐performance catalysts to the fields of TME‐modulating and multifunctional nanosystem‐based NCT for enhanced antitumor performance. Meanwhile, the novel nanocatalysts have gained more favorable biocompatibility, endowed with high chemical activity with relatively lower dosage, developing faster toward clinical application.^[^ ^63^ ^]^

Several researchers have made comprehensive summary on the progress of NCT in the past few years. Qiao and co‐workers.^[19b]^ provided a preliminary summary of the selection of Fenton/Fenton‐like nanozyme for NCT, while Zhang and co‐workers.^[19d]^ presented the approaches of modulation of TME which could improve the catalytic efficiency of nanozymes. With the in‐depth researches of NCT, the multifunctional catalytic nanosystems for biomedical applications emerged. Huo et al.^[19c]^ and Jia et al.^[19a]^ summarized the progress of NCT categorized by various nanozymes and modified nanosystems; the common and unique characteristics of these nanozymes were discussed to facilitate their biomedical application. Although detailed and elaborate information of NCT has been concluded by these reviews, the updated overview of the strategies on improving the effect of NCT was not well illustrated.

The present review provided updated insights into the novel strategies for optimized NCT. The comparisons of primary and updated strategies on NCT are illustrated in **Table** **1**. We summarized these strategies into two basic parts. Part 1 shows the improvement on the catalytic reaction, including high‐performance nanocatalysts and TME‐modulating approaches. Part 2 shows the design and construction of multifunctional nansystems for enhanced NCT against tumor, including targeting‐engineering, stimuli‐enhanced, synergistic, and theranostic applications. Part 1 is considered as the core of NCT, and the previous studies have dedicated to innovate desirable nanozymes and favorable TME for improved effect of NCT. In comparison, Part 2 is the extension of part 1 which is expected to be the focus in the next‐step research of NCT. It should be noted that although NCT was based on the in situ chemical reactions to kill cancer cells, it has limitations on nontargeting delivery, relatively low catalytic efficiency, and undetectable therapeutic process in vivo, which surmounted its further biomedical translation. The formulation of the multifunctional nansystems may tackle the above limitations and promote the further development of NCT. For example, with the development of molecular imaging, studies have achieved the real‐time monitoring of the NCT process by constructing nanozyme‐based systems. Upon the accurate diagnostic approaches involving high‐resolution PA, fluorescence, CT, and MRI imaging, clinicians would get timely feedback on the therapeutic process and effect, which have gained great potential for further clinical application.

**Table 1:** Summary of primary and updated strategies on optimizing NCT against tumors

Basic Parts of NCTa)		Primary strategies	Ref.	Updated strategies	Ref.
Catalytic reaction	Nanozyme	Fe, Cu, Mn, Ce, MOF‐based nanocomposites	[11]	Surface modification	[24]
				Morphological (2D) nanozyme	[26]
				SACs	[29]
	TME modulating	Co‐loaded catalysts to promote sequential catalytic reactions	[32, 34]	Self‐amplified H_2_O_2_ level	[35, 36]
				Improving the GSH/pH condition	[37, 40]
Construction of multifunctional nanosystems	Synergistic therapy	NCT+chemotherapy	[13]	NCT+stimuli–responsive therapy	[45, 46, 47, 48]
		NCT + PDT/ PTT	[44]	NCT+gene‐editing therapy	[50]
				NCT+immumotherapy	[53, 57]
	Targeted therapy	/		Vehicle mediated	[41]
				Surface ligand mediated	[43]
	Theranostic applications	/		Fluorescence imaging‐guided NCT	[59]
				PA‐guided NCT	[61]
				Multimodal imaging‐monitored NCT	[60]

a) MOF, metal–organic framwork; TME, tumor microenvironment; NCT, nanocatalytic therapy, PDT, photodynamic therapy; PTT, photothermal therapy; PA, photoacoustic.

**Figure 13:**
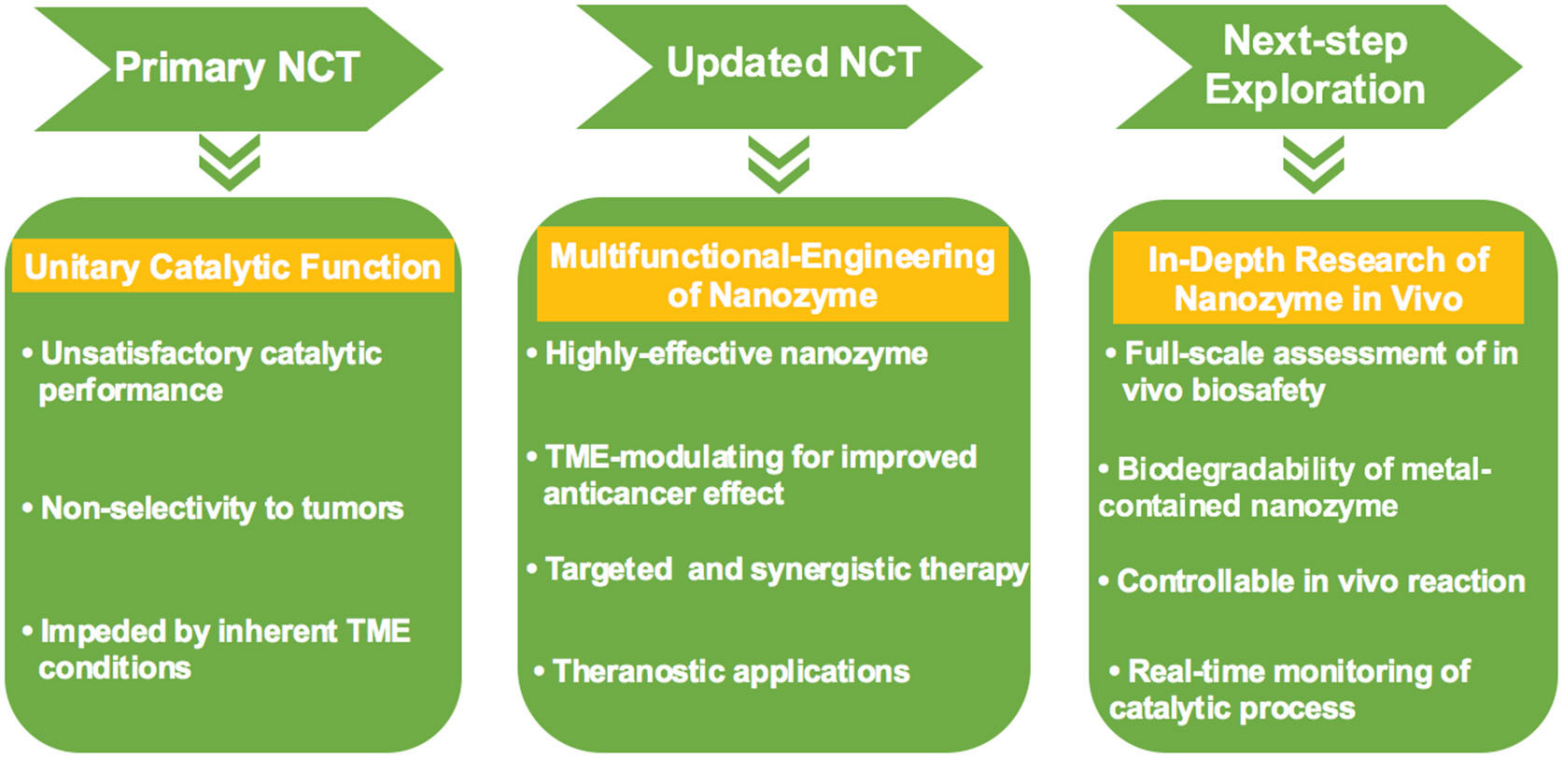
Summary of the development and current applications of nanozyme in cancer therapy, and future prospects of in‐depth research for further potential clinical translation.

On the other hand, although the present NCTs have exhibited remarkable antitumor outcomes, there are several obstacles to be addressed. 1) First, the biocompatibility, biodegradability, and metabolism of nanozymes in vivo are still intrinsic barriers for biomedical translation of NCT.^[^ ^64^ ^]^ Metal ion release of most nanozymes is considered as the possible factor to induce side effects in normal tissues due to the metal overload, which may produce ROS via Fenton/Fenton‐like reactions that destroy the biomacromolecules and necluic acids in normal tissues.^[19c]^ The recent studies proposed the strategy of surface modification to improve the biocompatibility of the nanozymes, which could also be excreted out of body by rapid kidney clearance, providing a feasible way to this issue.^[^ ^35^ ^]^ Full‐scale assessment of pharmacokinetics and metabolism of the nanozymes in vivo should be addressed in the next‐step research to ensure the biocompatibility and biosafety of NCT. 2) Second, the antitumor mechanism of NCT is preliminarily proved to be the apoptosis and necrosis of tumor cells induced by elevated ROS in TME. However, with the participation of metal ions, it becomes complicated to evaluate the deep‐seated mechanism of NCT on tumor cells, and the relevant research is lacking. Especially, with the emerging concept of ferroptosis^[10c]^ and cuproptosis,^[^ ^65^ ^]^ the completely new understandings of the antitumor mechanism of Fe/Cu ions were established. As such, the in‐depth studies of the molecular mechanism of NCT combating tumor cells should also be highlighted, which would promote the development of NCT on pharmacological research, targeted therapy, and further clinical application. 3) Third, the process of nanocatalytic reaction could be fully verified in vitro by enzymatic dynamic estimations. However, in vivo experiments, the characteristic parameters of NCT such as the reaction rate and concentrations of reactants, could not get timely recorded. The process and effect of NCT in vivo could only be preliminarily evaluated by the ROS‐responsive fluorescence imaging which is not favorable for the real‐time monitoring and controllability of the overall reaction. Novel accurate probes targeted to the nanocatalytic reactions were highly warranted to make the catalytic reaction measurable and controllable in vivo. 4) Last but not the least, at present, the majority of in vivo experiments of NCT utilized subcutaneous tumor models, with intratumoral or intravenous injection of nanozymes. The orthotopic tumor model in preferable accordance with clinical applications (biological, pathological and pharmacological features) should be established to further evaluate the effect and side effects of NCT. Moreover, it would be more favorable to explore targeted therapy and real‐time monitoring of NCT using orthotopic tumor model with higher reliability and tumor specificity, which may be of great value to facilitate clinical translation of NCT in the future (**Figure** **13**).

## Conflict of Interest

The authors declare no conflict of interest.

## Author Contributions

Z.L.L., D.S.H., and T.Y.: study concept and design; Z.L.L., H.W., J.Q.Z., and L.Y.S.: drafting of the manuscript; X.M.T., D.S.H., and T.Y.: administrative, technical, or material support; D.S.H., and T.Y.: critical revision; D.S.H., and T.Y.: study supervision.

## References

[1] a) R. L. Siegel , K. D. Miller , H. E. Fuchs , A. Jemal , CA Cancer J. Clin. 2021, 71, 7; 33433946 10.3322/caac.21654

[2] a) J. C. Dawson , A. Serrels , D. G. Stupack , D. D. Schlaepfer , M. C. Frame , Nat. Rev. Cancer 2021, 21, 313; 33731845 10.1038/s41568-021-00340-6 PMC8276817

[3] H. Lin , Y. Chen , J. Shi , Chem. Soc. Rev. 2018, 47, 1938. 29417106 10.1039/c7cs00471k

[4] a) G. Yang , L. Xu , Y. Chao , J. Xu , X. Sun , Y. Wu , R. Peng , Z. Liu , Nat. Commun. 2017, 8, 902; 29026068 10.1038/s41467-017-01050-0 PMC5638920

[5] a) T. Finkel , M. Serrano , M. A. Blasco , Nature 2007, 448, 767; 17700693 10.1038/nature05985

[6] a) S. Wang , H. Zheng , L. Zhou , F. Cheng , Z. Liu , H. Zhang , L. Wang , Q. Zhang , Nano Lett. 2020, 20, 5149; 32574064 10.1021/acs.nanolett.0c01371

[7] L. Gao , J. Zhuang , L. Nie , J. Zhang , Y. Zhang , N. Gu , T. Wang , J. Feng , D. Yang , S. Perrett , X. Yan , Nat. Nanotechnol. 2007, 2, 577. 18654371 10.1038/nnano.2007.260

[8] a) B. Yang , Y. Chen , J. Shi , Adv. Mater. 2019, 31, 1901778;

[9] a) L. Zhu , Y. Dai , L. Gao , Q. Zhao , Int. J. Nanomedicine 2021, 16, 4559; 34267513 10.2147/IJN.S309062 PMC8275154

[10] X. Mei , T. Hu , H. Wang , R. Liang , W. Bu , M. Wei , Biomaterials 2020, 258, 120257. 32798739 10.1016/j.biomaterials.2020.120257

[11] a) L. Zeng , H. Cheng , Y. Dai , Z. Su , C. Wang , L. Lei , D. Lin , X. Li , H. Chen , K. Fan , S. Shi , ACS Appl. Mater. Interfaces 2021, 13, 233; 33373178 10.1021/acsami.0c19074

[12] a) S. Sheng , F. Liu , L. Lin , N. Yan , Y. Wang , C. Xu , H. Tian , X. Chen , J Control Release 2020, 328, 631; 32950593 10.1016/j.jconrel.2020.09.029

[13] L. Wang , J. Xia , H. Fan , M. Hou , H. Wang , X. Wang , K. Zhang , L. Cao , X. Liu , J. Ling , H. Yu , X. Wu , J. Sun , Theranostics 2021, 11, 8909. 34522218 10.7150/thno.61651 PMC8419042

[14] J. Wang , D. Wang , M. Cen , D. Jing , J. Bei , Y. Huang , J. Zhang , B. Lu , Y. Wang , Y. Yao , J. Nanobiotechnol. 2022, 20, 33. 10.1186/s12951-021-01237-0 PMC8753913 35016673

[15] M. Wang , M. Chang , C. Li , Q. Chen , Z. Hou , B. Xing , J. Lin , Adv. Mater. 2022, 34, 2106010. 10.1002/adma.202106010 34699627

[16] C. Xin , Y. Zhang , M. Bao , C. Yu , K. Hou , Z. Wang , J Colloid Interface Sci. 2022, 606, 1488. 34500153 10.1016/j.jcis.2021.08.121

[17] L. Ming , L. Song , J. Xu , R. Wang , J. Shi , M. Chen , Y. Zhang , ACS Appl. Mater. Interfaces 2021, 13, 35444. 34292714 10.1021/acsami.1c08927

[18] Y. Li , J. Yang , G. Gu , X. Guo , C. He , J. Sun , H. Zou , H. Wang , S. Liu , X. Li , S. Zhang , K. Wang , L. Yang , Y. Jiang , L. Wu , X. Sun , Nano Lett. 2022, 22, 963. 35073699 10.1021/acs.nanolett.1c03786

[19] a) C. Jia , Y. Guo , F. G. Wu , Small. 2022, 18, 2103868; 10.1002/smll.202103868 34729913

[20] Z. Chen , J. J. Yin , Y. T. Zhou , Y. Zhang , L. Song , M. Song , S. Hu , N. Gu , ACS Nano. 2012, 6, 4001. 22533614 10.1021/nn300291r

[21] a) C. Tapeinos , A. Pandit , Adv. Mater. 2016, 28, 5553; 27184711 10.1002/adma.201505376

[22] Y. Huang , J. Ren , X. Qu , Chem. Rev. 2019, 119, 4357. 30801188 10.1021/acs.chemrev.8b00672

[23] P. Wang , T. Wang , J. Hong , X. Yan , M. Liang , Front. Bioeng. Biotechnol. 2020, 8, 15. 32117909 10.3389/fbioe.2020.00015 PMC7015899

[24] R. Yang , S. Fu , R. Li , L. Zhang , Z. Xu , Y. Cao , H. Cui , Y. Kang , P. Xue , Theranostics 2021, 11, 107. 33391464 10.7150/thno.50486 PMC7681078

[25] a) N. Y. Kim , S. Blake , D. De , J. Ouyang , J. Shi , N. Kong , Front. Pharmacol. 2019, 10, 1573; 32038249 10.3389/fphar.2019.01573 PMC6985776

[26] F. Gong , N. Yang , Y. Wang , M. Zhuo , Q. Zhao , S. Wang , Y. Li , Z. Liu , Q. Chen , L. Cheng , Small 2020, 16, 2003496. 10.1002/smll.202003496 33107203

[27] a) X. Lu , S. Gao , H. Lin , J. Shi , Small 2021, 17, 2004467; 10.1002/smll.202004467 33448133

[28] a) H. Xiang , W. Feng , Y. Chen , Adv. Mater. 2020, 32, 1905994; 10.1002/adma.201905994 31930751

[29] X. Lu , S. Gao , H. Lin , L. Yu , Y. Han , P. Zhu , W. Bao , H. Yao , Y. Chen , J. Shi , Adv. Mater. 2020, 32, 2002246. 10.1002/adma.202002246 32705751

[30] J. Yang , H. Yao , Y. Guo , B. Yang , J. Shi , Angew. Chem. Int. Ed. Engl. 2022, 61, 202200480. 10.1002/anie.202200480 35143118

[31] a) M. H. Raza , S. Siraj , A. Arshad , U. Waheed , F. Aldakheel , S. Alduraywish , M. Arshad , J. Cancer Res. Clin. Oncol. 2017, 143, 1789; 28647857 10.1007/s00432-017-2464-9 PMC11819417

[32] a) Q. Chen , C. Liang , X. Sun , J. Chen , Z. Yang , H. Zhao , L. Feng , Z. Liu , Proc. Natl. Acad. Sci. U.S.A. 2017, 114, 5343; 28484000 10.1073/pnas.1701976114 PMC5448233

[33] N. A. Kotov , Science 2010, 330, 188. 20929766 10.1126/science.1190094

[34] S. Gao , H. Lin , H. Zhang , H. Yao , Y. Chen , J. Shi , Adv. Sci. 2019, 6, 1801733. 10.1002/advs.201801733 PMC6364502 31168441

[35] K. Yang , G. Yu , Z. Yang , L. Yue , X. Zhang , C. Sun , J. Wei , L. Rao , X. Chen , R. Wang , Angew. Chem. Int. Ed. Engl. 2021, 60, 17570. 34041833 10.1002/anie.202103721

[36] Y. Sang , F. Cao , W. Li , L. Zhang , Y. You , Q. Deng , K. Dong , J. Ren , X. Qu , J. Am . Chem. Soc. 2020, 142, 5177. 10.1021/jacs.9b12873 32100536

[37] S. Fu , R. Yang , L. Zhang , W. Liu , G. Du , Y. Cao , Z. Xu , H. Cui , Y. Kang , P. Xue , Biomaterials 2020, 257, 120279. 32763613 10.1016/j.biomaterials.2020.120279

[38] a) C. Xu , W. Bing , F. Wang , J. Ren , X. Qu , ACS Nano 2017, 11, 7770; 28661119 10.1021/acsnano.7b01450

[39] a) E. G. Heckert , S. Seal , W. T. Self , Environ. Sci. Technol. 2008, 42, 5014; 18678042 10.1021/es8001508 PMC3036004

[40] S. Dong , Y. Dong , T. Jia , S. Liu , J. Liu , D. Yang , F. He , S. Gai , P. Yang , J. Lin , Adv. Mater. 2020, 32, 2002439. 10.1002/adma.202002439 32914495

[41] H. Wu , H. Xing , M. C. Wu , F. Shen , Y. Chen , T. Yang , Theranostics 2021, 11, 64. 33391461 10.7150/thno.46124 PMC7681081

[42] a) G. Chen , A. C. Huang , W. Zhang , G. Zhang , M. Wu , W. Xu , Z. Yu , J. Yang , B. Wang , H. Sun , H. Xia , Q. Man , W. Zhong , L. F. Antelo , B. Wu , X. Xiong , X. Liu , L. Guan , T. Li , S. Liu , R. Yang , Y. Lu , L. Dong , S. McGettigan , R. Somasundaram , R. Radhakrishnan , G. Mills , Y. Lu , J. Kim , Y. H. Chen , H. Dong , Y. Zhao , G. C. Karakousis , T. C. Mitchell , L. M. Schuchter , M. Herlyn , E. J. Wherry , X. Xu , W. Guo , Nature 2018, 560, 382; 30089911 10.1038/s41586-018-0392-8 PMC6095740

[43] Z. Wang , Z. Li , Z. Sun , S. Wang , Z. Ali , S. Zhu , S. Liu , Q. Ren , F. Sheng , B. Wang , Y. Hou , Sci. Adv. 2020, 6, eabc8733. 33246959 10.1126/sciadv.abc8733 PMC7695480

[44] a) Y. Li , R. Zhang , Q. Wan , R. Hu , Y. Ma , Z. Wang , J. Hou , W. Zhang , B. Z. Tang , Adv. Sci. 2021, 8, 2102561; 10.1002/advs.202102561 PMC8655165 34672122

[45] J. Liu , A. Wang , S. Liu , R. Yang , L. Wang , F. Gao , H. Zhou , X. Yu , J. Liu , C. Chen , Angew. Chem. Int. Ed. Engl. 2021, 60, 25328. 34453387 10.1002/anie.202106750

[46] W. Wu , Y. Pu , J. Shi , Adv. Sci. 2021, 8, 2002816. 10.1002/advs.202002816 PMC8097343 33977044

[47] a) Y. Du , X. Liu , Q. Liang , X. J. Liang , J. Tian , Nano Lett. 2019, 19, 3618; 31074627 10.1021/acs.nanolett.9b00630

[48] Y. Zhang , X. Wang , C. Chu , Z. Zhou , B. Chen , X. Pang , G. Lin , H. Lin , Y. Guo , E. Ren , P. Lv , Y. Shi , Q. Zheng , X. Yan , X. Chen , G. Liu , Nat. Commun. 2020, 11, 5421. 33110072 10.1038/s41467-020-19061-9 PMC7591490

[49] a) J. Guo , Z. Yu , M. Das , L. Huang , ACS Nano 2020, 14, 5075; 32283007 10.1021/acsnano.0c01676

[50] C. Liu , Y. Chen , J. Zhao , Y. Wang , Y. Shao , Z. Gu , L. Li , Y. Zhao , Angew. Chem. Int. Ed. Engl. 2021, 60, 14324. 33822451 10.1002/anie.202101744

[51] a) M. Song , T. Liu , C. Shi , X. Zhang , X. Chen , ACS Nano 2016, 10, 633; 26650065 10.1021/acsnano.5b06779 PMC5242343

[52] a) D. M. Mosser , J. P. Edwards , Nat. Rev. Immunol. 2008, 8, 958; 19029990 10.1038/nri2448 PMC2724991

[53] B. Xu , Y. Cui , W. Wang , S. Li , C. Lyu , S. Wang , W. Bao , H. Wang , M. Qin , Z. Liu , W. Wei , H. Liu , Adv. Mater. 2020, 32, 2003563. 10.1002/adma.202003563 32627937

[54] S. Srivastava , S. N. Furlan , C. A. Jaeger-Ruckstuhl , M. Sarvothama , C. Berger , K. S. Smythe , S. M. Garrison , J. M. Specht , S. M. Lee , R. A. Amezquita , V. Voillet , V. Muhunthan , S. Yechan-Gunja , S. Pillai , C. Rader , A. M. Houghton , R. H. Pierce , R. Gottardo , D. G. Maloney , S. R. Riddell , Cancer Cell. 2021, 39, 193. 33357452 10.1016/j.ccell.2020.11.005 PMC7878409

[55] H. Li , Y. Huang , D. Q. Jiang , L. Z. Cui , Z. He , C. Wang , Z. W. Zhang , H. L. Zhu , Y. M. Ding , L. F. Li , Q. Li , H. J. Jin , Q. J. Qian , Cell Death Dis. 2018, 9, 177. 29415996 10.1038/s41419-017-0238-6 PMC5833445

[56] H. He , Z. Fei , T. Guo , Y. Hou , D. Li , K. Wang , F. Ren , K. Fan , D. Zhou , C. Xie , C. Wang , X. Lu , Biomaterials. 2022, 280, 121272. 34864428 10.1016/j.biomaterials.2021.121272

[57] L. Zhu , J. Liu , G. Zhou , T. M. Liu , Y. Dai , G. Nie , Q. Zhao , Small. 2021, 17, 2102624. 10.1002/smll.202102624 34378338

[58] a) S. Hapuarachchige , D. Artemov , Front. Oncol. 2020, 10, 1131; 32793481 10.3389/fonc.2020.01131 PMC7387661

[59] T. Chen , P. Hou , Y. Zhang , R. Ao , L. Su , Y. Jiang , Y. Zhang , H. Cai , J. Wang , Q. Chen , J. Song , L. Lin , H. Yang , X. Chen , Angew. Chem. Int. Ed. Engl. 2021, 60, 15006. 33871140 10.1002/anie.202102097

[60] Z. Zheng , Z. Jia , Y. Qin , R. Dai , X. Chen , Y. Ma , X. Xie , R. Zhang , Small 2021, 17, 2103252. 10.1002/smll.202103252 34499414

[61] S. Lei , J. Zhang , N. T. Blum , M. Li , D. Y. Zhang , W. Yin , F. Zhao , J. Lin , P. Huang , Nat. Commun. 2022, 13, 1298. 35277519 10.1038/s41467-022-29082-1 PMC8917194

[62] a) X. Qian , J. Zhang , Z. Gu , Y. Chen , Biomaterials 2019, 211, 1; 31075521 10.1016/j.biomaterials.2019.04.023

[63] a) Z. Wang , Y. Zhang , E. Ju , Z. Liu , F. Cao , Z. Chen , J. Ren , X. Qu , Nat. Commun. 2018, 9, 3334; 30127408 10.1038/s41467-018-05798-x PMC6102211

[64] H. Wu , F. Chen , C. You , Y. Zhang , B. Sun , Q. Zhu , Small. 2020, 16, 2001805. 10.1002/smll.202001805 33079449

[65] D. Tang , X. Chen , G. Kroemer , Cell Res. 2022, 32, 417. 35354936 10.1038/s41422-022-00653-7 PMC9061796

